# A comparative analysis of risk stratification tools in SSc-associated pulmonary arterial hypertension: a EUSTAR analysis

**DOI:** 10.1093/rheumatology/keaf053

**Published:** 2025-01-29

**Authors:** Hilde Jenssen Bjørkekjær, Cosimo Bruni, Kaspar Broch, Cathrine Brunborg, Patricia E Carreira, Paolo Airò, Carmen Pilar Simeón-Aznar, Marie-Elise Truchetet, Alessandro Giollo, Alexandra Balbir-Gurman, Mickael Martin, Christopher P Denton, Armando Gabrielli, Francesco Del Galdo, Madelon C Vonk, Håvard Fretheim, Helle Bitter, Øyvind Midtvedt, Arne Andreassen, Sverre Høie, Yoshiya Tanaka, Gabriela Riemekasten, Ulf Müller-Ladner, Marco Matucci-Cerinic, Ivan Castellví, Elise Siegert, Eric Hachulla, Øyvind Molberg, Oliver Distler, Anna-Maria Hoffmann-Vold, EUSTAR Collaborators, Serena Guiducci, Florenzo Iannone, Simona Rednic, Yannick Allanore, Carlomaurizio Montecucco, Gábor Kumánovics, Michele Iudici, Gianluca Moroncini, Kristofer Andréasson, Luca Idolazzi, Jörg Henes, Johannes Pflugfelder, José António Pereira da Silva, Michael Hughes, Valeria Riccieri, Andra Balanescu, Ana Maria Gheorghiu, Christina Bergmann, Francesco Paolo Cantatore, Ellen De Langhe, Branimir Ani, Carolina de Souza Müller, Kamal Solanki, Edoardo Rosato, Britta Maurer, Lesley Ann Saketkoo, Massimiliano Limonta, Vivien M Hsu, Lorinda S Chung, Yair Levy, Petros Sfikakis, Susana Oliveira, Masataka Kuwana

**Affiliations:** Department of Rheumatology, Hospital of Southern Norway, Kristiansand, Norway; Institute of Clinical Medicine, University of Oslo, Oslo, Norway; Department of Rheumatology, University Hospital Zurich, University of Zurich, Zurich, Switzerland; Department of Experimental and Clinical Medicine, University of Florence, Florence, Italy; Department of Cardiology, Oslo University Hospital, Rikshospitalet, Oslo, Norway; Institute for Experimental Medical Research, KG Jebsen Center, University of Oslo, Oslo, Norway; Institute of Clinical Medicine, University of Oslo, Oslo, Norway; Oslo Centre for Biostatistics and Epidemiology, Research Support Services, Oslo University Hospital—Rikshospitalet, Oslo, Norway; Department of Rheumatology, 12 de Octubre University Hospital, Madrid, Spain; UOC Reumatologia ed Immunologia Clinica, 9 Spedali Civili di Brescia, Scleroderma UNIT, Brescia, Italy; Systemic Autoimmune Diseases Unit, Department of Internal Medicine, University Vall d'Hebron Hospital, Barcelona, Spain; Rheumatology Department, Bordeaux University Hospital, Bordeaux, France; Division of Rheumatology, Department of Medicine—DIMED, University and Hospital of Padua, Padova, Italy; Rheumatology Section, Department of Medicine, University of Verona, Verona, Italy; Rappaport Faculty of Medicine, Rheumatology Institute, Rambam Health Care Campus, Technion-Institute of Technology, Haifa, Israel; Department of Internal Medicine, Poitiers University Hospital, Poitiers, France; Centre for Rheumatology and Connective Tissue Diseases, University College London Division of Medicine and Royal Free Hospital, London, UK; Fondazione di Medicina Molecolare e Terapia Cellulare, Università Politecnica delle Marche, Ancona, Italy; Leeds Institute of Rheumatic and Musculoskeletal Medicine, LIRMM, Leeds, UK; NIHR Leeds Biomedical Research Centre, Leeds Teaching Hospitals Trust, Leeds, UK; Department of Rheumatology, Radboud Universiteit, Nijmegen, Netherlands; Institute of Clinical Medicine, University of Oslo, Oslo, Norway; Lillehammer Hospital for Rheumatic Diseases, Lillehammer, Norway; Department of Rheumatology, Hospital of Southern Norway, Kristiansand, Norway; Department of Rheumatology, Oslo University Hospital, Rikshospitalet, Oslo, Norway; Department of Cardiology, Oslo University Hospital, Rikshospitalet, Oslo, Norway; Institute of Clinical Medicine, University of Oslo, Oslo, Norway; Department of Cardiology, Hospital of Southern Norway, Arendal, Norway; First Department of Internal Medicine, University of Occupational and Environmental Health, Kitakyushu, Japan; Department of Rheumatology and Clinical Immunology, University of Lübeck, Lübeck, Germany; Department of Rheumatology and Clinical Immunology, Justus-Liebig University Giessen, Campus Kerckhoff, Bad Nauheim, Germany; Unit of Immunology, Rheumatology, Allergy and Rare Diseases (UnIRAR) & Inflammation, fibrosis and ageing initiative (INFLAGE), IRCCS San Raffaele Hospital, Milan, Italy; University Vita Salute San Raffaele, Milano, Italy; Department of Rheumatology, Hospital de la Santa Creu i Sant Pau, Barcelona, Spain; Rheumatology, Charite University Hospital, Berlin, Germany; Department of Internal Medicine and Clinical Immunology, Referral Centre for Centre for Rare Systemic Autoimmune Diseases North of France, North-West, Mediterranean and Guadeloupe (CeRAINOM), CHU Lille, Univ. Lille, Inserm, U1286 – INFINITE – Institute for Translational Research in Inflammation, Lille, France; Institute of Clinical Medicine, University of Oslo, Oslo, Norway; Department of Rheumatology, Oslo University Hospital, Rikshospitalet, Oslo, Norway; Department of Rheumatology, University Hospital Zurich, University of Zurich, Zurich, Switzerland; Department of Rheumatology, University Hospital Zurich, University of Zurich, Zurich, Switzerland; Department of Rheumatology, Oslo University Hospital, Rikshospitalet, Oslo, Norway

**Keywords:** observational study, pulmonary arterial hypertension, risk stratification, systemic sclerosis

## Abstract

**Objectives:**

The 2022 European Society of Cardiology and European Respiratory Society (ESC/ERS) guidelines for pulmonary arterial hypertension (PAH) recommend risk stratification to optimize management. However, the performance of generic PAH risk stratification tools in patients with SSc-associated PAH remains unclear. Our objective was to identify the most accurate approach for risk stratification at SSc-PAH diagnosis.

**Methods:**

In this multicentre, international cohort study from the European Scleroderma Trials and Research (EUSTAR) group database, we screened 11 risk stratification tools upon SSc-PAH diagnosis. We compared the performance of the three top-ranked tools to predict mortality with the ESC/ERS three-strata model, the currently recommended tool for baseline risk assessment. We also assessed the impact of incorporating SSc-specific characteristics into the tools. Kaplan–Meier analyses and Cox regression with area under the ROC curve (AUC) were conducted.

**Results:**

The ESC/ERS three-strata model had a lower ability to predict mortality than the ESC/ERS four-strata model, ‘SPAHR updated’ and ‘REVEAL Lite 2’. The ESC/ERS four-strata model divided ‘intermediate-risk’ patients into two groups with significantly different long-term survival rates and is the easiest applicable tool. Incorporating SSc-specific characteristics did not significantly improve the predictive ability of any model, but a low diffusing capacity of the lung for carbon monoxide (DLCO) was an independent predictor of mortality.

**Conclusion:**

Considering its ability to predict mortality, risk segregation capabilities and clinical applicability, this study provides a rationale for using the simplified ESC/ERS four-strata model at SSc-PAH diagnosis as an alternative to the comprehensive ESC/ERS three-strata model. We propose considering DLCO as an individual prognostic marker in SSc-PAH.

## Graphical abstract

**Figure:**
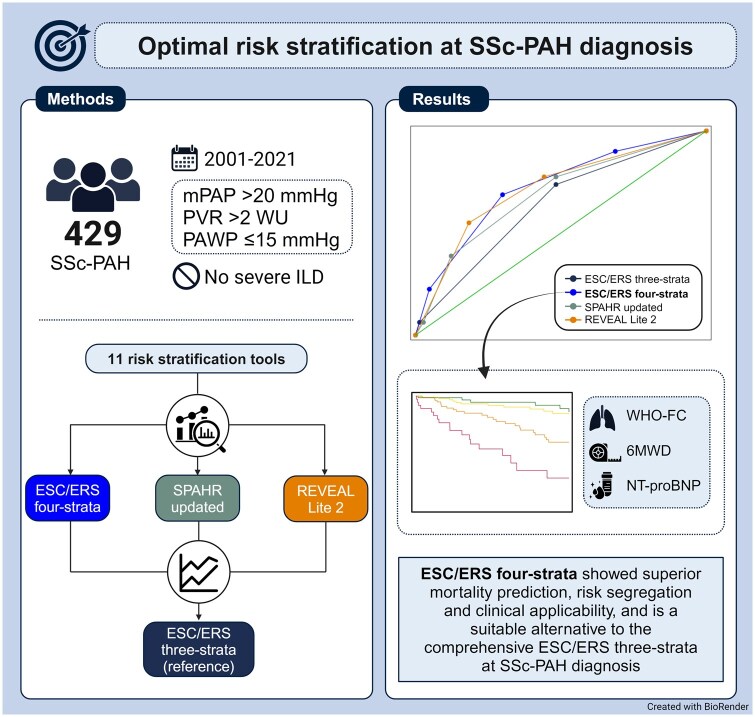

Rheumatology key messages

- The ESC/ERS four-strata model showed superior mortality prediction, risk segregation and applicability at SSc-PAH diagnosis.
- Incorporating SSc-specific characteristics did not improve predictive accuracy, but DLCO was an independent prognostic marker.
- Risk stratification was accurate in all SSc-PAH patients, regardless of pre-existing vascular-targeted therapies and haemodynamic thresholds.

## Introduction

Pulmonary arterial hypertension (PAH) develops in 6–12% of patients with SSc [1–3]. Despite often presenting with milder haemodynamic impairment, patients with SSc-PAH have a worse prognosis and respond less favourably to treatment compared with those with idiopathic PAH (IPAH) [4–7]. This may be attributed to the heterogeneity and complexity of SSc, including diverse pathogenic mechanisms and systemic organ involvement, which may lead to multiple mechanisms contributing to pulmonary hypertension [6, 8–10]. In recent years, studies suggest an improvement in the survival of patients diagnosed with SSc-PAH [11, 12], possibly due to enhanced screening, earlier diagnosis and novel treatment strategies [11–18].

The 2022 European Society of Cardiology and European Respiratory Society (ESC/ERS) guidelines for PAH, along with the updates from the Seventh World Symposium on Pulmonary Hypertension (WSPH), recommend risk stratification to predict mortality risk and guide treatment decisions [19, 20]. To assess the baseline risk, the guidelines recommend the comprehensive ESC/ERS three-strata model, which combines up to 18 risk parameters to define low-, intermediate- or high-risk status with estimated 1-year mortality rates of <5%, 5–20% and >20%, respectively [19]. At follow-up, a simplified four-strata model based on WHO functional class (WHO-FC), 6-min walk distance (6MWD) and brain natriuretic peptide (BNP) or N-terminal (NT)-proBNP is recommended [19, 21]. Several other risk stratification tools have been proposed [21–29], predominantly developed using data from patients with IPAH, thus not considering the distinctive characteristics of SSc-PAH, such as multiorgan involvement and potential unique prognostic markers.

Our objective was to identify the most accurate approach for risk stratification in SSc-PAH at the time of diagnosis by comparing various tools to the ESC/ERS three-strata model, the currently recommended tool for baseline risk assessment, and to assess the impact of incorporating SSc-specific characteristics to improve the accuracy of these tools.

## Study design and methods

### Study design

This multicentre, international cohort study included all SSc-PAH patients in the European Scleroderma Trials and Research (EUSTAR) database with right heart catheterizations (RHCs) and annual prospective data, extracted on 1 April 2022. Additional data were collected via specific case report forms through direct contact with the centres. The database structure has been previously described [30]. The project was approved by the EUSTAR board (project number: CP122). The study complies with the Declaration of Helsinki. Each participating centre obtained approval from the local ethics committee. The coordinating centre’s protocol was approved by the Regional Committees for Medical and Health Research Ethics (REK) in Norway (approval number: 273870).

### Study subjects and inclusion criteria

We assessed patients who had at least one RHC between 2001 and 2021 and met the following criteria: (i) 2022 haemodynamic definition of PAH (mean pulmonary arterial pressure [mPAP] >20 mmHg, pulmonary artery wedge pressure [PAWP] ≤15 mmHg and pulmonary vascular resistance [PVR] >2 Wood Units [WUs]) [19]; (ii) age ≥18 years and (iii) 2013 ACR/EULAR SSc classification criteria [31]. Patients with severe interstitial lung disease (ILD), defined as an extent of ILD on high-resolution computed tomography (HRCT) >20% or a forced vital capacity (FVC) <70% in the presence of ILD, without available quantification, were excluded [32]. We recorded demographic and clinical characteristics at RHC. SSc disease duration was defined as the time from the first non-Raynaud symptom to RHC. Treatment-naïve status was defined as no pre-existing therapies targeting vascular symptoms such as Raynaud phenomenon or digital ulcers (DUs) (e.g. endothelin receptor antagonists [ERAs], phosphodiesterase-5 inhibitors [PDE-5is] or prostacyclin pathway agents [PPAs]). Initial treatment strategies were defined as (i) upfront monotherapy with ERA, PDE-5i (including soluble guanylate cyclase stimulator), or PPA; or (ii) upfront dual or triple combination therapy with these drugs within four months of PAH diagnosis. Higher and lower mPAP and PVR threshold groups were defined according to the 2015 and 2022 haemodynamic criteria: mPAP ≥25 mmHg and PVR >3 WU, and mPAP 21–24 mmHg or PVR 2–3 WU, respectively.

### Outcomes

**Figure 1.**
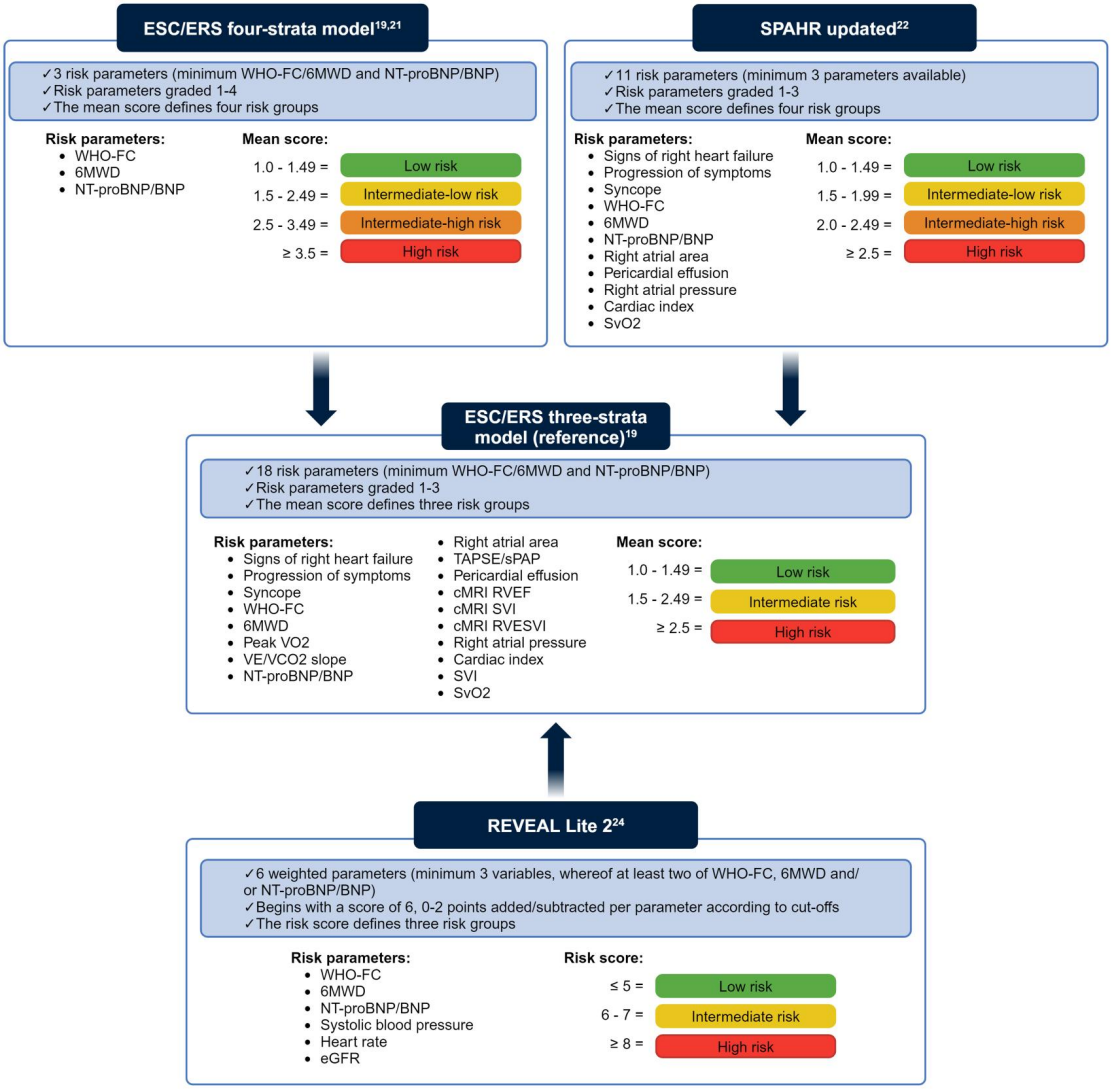
Description of the risk stratification tools and calculation of risk scores. ESC/ERS: European Society of Cardiology and European Respiratory Society; SPAHR: Swedish Pulmonary Arterial Hypertension Registry; REVEAL: Registry to Evaluate Early and Long-Term PAH Disease Management; WHO-FC: World Health Organization functional class; 6MWD: 6-min walk distance; NT-proBNP: N-terminal brain natriuretic peptide; SvO_2_: mixed-venous oxygen saturation; VO_2_: oxygen uptake; VE/VCO_2_: ventilatory equivalents for carbon dioxide; TAPSE/sPAP: tricuspid annular plane systolic excursion/systolic pulmonary artery pressure; cMRI: cardiac magnetic resonance imaging; RVEF: right ventricular ejection fraction; SVI: stroke volume index; RVESVI: right ventricular end-systolic volume index; eGFR: estimated glomerular filtration rate. The figure was created using BioRender.com

The primary outcome was all-cause mortality, defined from the date of SSc-PAH diagnosis by RHC until death, or the censoring date (lung transplantation or study end, defined as the date last known to be alive). We conducted a two-stage evaluation of generic PAH risk stratification tools, using the ESC/ERS three-strata model as the reference. First, we ranked these tools based on their applicability and performance in predicting mortality in the SSc-PAH cohort. To maintain a clear and focused analysis, we selected the three top performing tools for comparison against the reference (Fig. 1). We followed the ESC/ERS three-strata model guidelines, incorporating as many risk parameters as possible, including at least WHO-FC or 6MWD and BNP or NT-proBNP [19]. In the absence of a validated method for calculating a risk score, we applied an approach proposed by previous studies, assigning scores to parameters based on cut-off thresholds provided in the guidelines, with the mean score determining the risk category: <1.50 (low risk), 1.50–2.49 (intermediate risk) and ≥2.50 (high risk) [27, 28].

We evaluated the distribution of risk groups and compared observed 1-year mortality with expected mortality as estimated by the guidelines. We assessed transplant-free survival (TFS) by risk groups and compared each tool’s ability to predict all-cause mortality against the ESC/ERS three-strata reference tool. Finally, we tested the impact of incorporating SSc-specific characteristics into the risk stratification tools, including predictors of outcomes in SSc, based on previous studies and expert opinions of the co-authors [32–40]. The final covariates for the multivariable models were selected through an evaluation of variable availability, multicollinearity, and model performance.

The outcome was assessed in all SSc-PAH patients and in predefined subgroup analyses: PAH treatment-naïve patients, patients categorized by haemodynamic thresholds and those meeting all risk stratification tool criteria.

### Statistics

Statistical analyses were performed with IBM SPSS, version 29, and STATA, version 18. Categorical variables were compared using Pearson χ^2^ or Fisher exact test, and continuous variables with independent sample *t* test or Mann–Whitney *U* test, as appropriate. TFS was evaluated using Kaplan–Meier analysis and the log-rank test. Univariable and multivariable Cox regression models assessed the risk stratification tools’ ability to predict all-cause mortality, presenting hazard ratios (HRs) and 95% CIs. Multicollinearity was evaluated using Pearson’s and Spearman’s correlation coefficients, with a cut-off of ≥0.7. Multivariable models required 10 outcome events per covariate. The predictive ability of the tools was compared using area under the ROC curve (AUC).

Sensitivity analyses were performed with multiple imputations for missing covariates in the multivariable regression model, except for the risk parameters, which were treated as the exposure variable in the analyses. Under the assumption of missing at random, 40 imputed datasets were generated using the multiple imputation chained procedure in STATA. Multivariable regression analyses were repeated across these datasets, with results pooled using Rubin’s rules.

## Results

### Baseline characteristics

Of the 889 SSc patients in the EUSTAR database with RHC, 429 SSc-PAH patients from 43 centres were eligible (Supplementary Fig. S1, available at *Rheumatology* online). Among these, 288 (67%) were treatment-naïve, and 141 (33%) had pre-existing vascular-targeted therapies (Table 1). Treatment-naïve patients had shorter SSc disease duration, lower prevalence of DUs, higher diffusing capacity of the lung for carbon monoxide (DLCO), smaller right atrial area, higher occurrence of diastolic dysfunction and higher frequency of initiating upfront PAH therapy (Table 1). Over a median follow-up of 3.3 years (Q1–Q3: 1.4–5.6), 172 (40%) of the SSc-PAH patients died, and 14 (4%) underwent lung transplantation. The overall 1-, 3- and 5-year TFS rates were 93%, 78% and 64%, respectively. Treatment-naïve patients had a better long-term survival rate compared with those receiving pre-existing treatment (Table 1 and Supplementary Fig. S2, available at *Rheumatology* online). There were no significant differences in survival according to the diagnostic period before and after 2015 (Supplementary Fig. S3, available at *Rheumatology* online).

**Table 1.** Comparison of baseline characteristics in treatment-naïve patients *vs* patients with pre-existing vascular-targeted therapies

	No.	All SSc-PAH (*n* = 429)	Treatment-naïve (*n* = 288)	Pre-existing treatment (*n* = 141)	*P*
Age, years (SD)	429	65 ± 11	66 ± 11	65 ± 11	0.753
Male sex, no. (%)	429	60 (14.0)	37 (12.9)	23 (16.3)	0.331
SSc characteristics					
SSc duration, years (Q1–Q3)	406	9.7 (3.7**–**16.5)	8.5 (2.4**–**15.5)	12.7 (5.5**–**19.3)	0.0008
lcSSc, no. (%)	420	342 (81.4)	235 (83.9)	107 (76.4)	0.062
mRSS, mean (SD)	361	4.4 ± 6.2	4.5 ± 6.3	4.2 ± 6.0	0.659
ACA positive, no. (%)	427	273 (63.9)	181 (63.1)	92 (65.7)	0.593
Digital ulcers, no. (%)	423	170 (40.2)	97 (34.0)	73 (52.9)	<0.001
Telangiectasia, no. (%)	417	352 (84.4)	236 (83.7)	116 (85.9)	0.556
Joint synovitis, no. (%)	387	57 (14.7)	36 (14.2)	21 (15.7)	0.703
Muscle weakness, no. (%)	359	60 (16.7)	30 (12.9)	30 (23.8)	0.008
Renal crisis, no. (%)	407	16 (3.9)	13 (4.7)	3 (2.3)	0.241
Lung characteristics					
FVC, % predicted (SD)	408	91.3 ± 21.1	90.8 ± 20.1	92.3 ± 23.0	0.485
DLCO, % predicted (Q1–Q3)	382	43 (33**–**52)	45 (34**–**53)	40 (33**–**50)	0.038
6MWD, m (SD)	306	341 ± 127	342 ± 130	340 ± 121	0.930
WHO-FC III and IV, no. (%)	418	211 (50.5)	142 (50.4)	69 (50.7)	0.942
ILD, no. (%)	429	187 (43.6)	130 (45.1)	57 (40.4)	0.355
Heart characteristics					
NT-proBNP, ng/L (Q1–Q3)	260	568 (203**–**1495)	623 (211**–**1599)	490 (176**–**1260)	0.467
Right atrial area, cm^2^ (Q1–Q3)	111	17.6 (14.9**–**22.0)	16.8 (14.0**–**20.1)	20.5 (17.4**–**24.8)	0.036
Pericardial effusion, no. (%)	379	65 (17.2)	44 (16.5)	21 (18.8)	0.593
TAPSE/sPAP, mm/mmHg (Q1–Q3)	166	0.36 (0.23**–**0.49)	0.33 (0.22**–**0.48)	0.40 (0.25**–**0.50)	0.169
Diastolic dysfunction, no. (%)	300	132 (44.0)	97 (49.5)	35 (33.7)	0.009
mPAP, mmHg (Q1–Q3)	429	33 (26**–**43)	32 (26**–**44)	34 (27**–**42)	0.479
PAWP, mmHg (Q1–Q3)	429	9 (7**–**12)	10 (7**–**12)	9 (7**–**12)	0.893
PVR, WU (Q1–Q3)	429	5.3 (3.3**–**8.0)	5.1 (3.2**–**7.9)	5.6 (3.4**–**8.1)	0.255
CI, L/min/m^2^ (Q1–Q3)	398	2.7 (2.2**–**3.2)	2.7 (2.2**–**3.2)	2.7 (2.2**–**3.2)	0.774
Lower mPAP/PVR, no. (%)	429	118 (27.5)	85 (29.5)	33 (23.4)	0.183
Other characteristics					
Upfront treatment, no. (%)	422	245 (58.1)	183 (65.1)	62 (44.0)	<0.001
• Monotherapy, no. (%)	422	159 (37.7)	108 (38.4)	51 (36.2)	0.651
• Combination, no. (%)	422	86 (20.4)	75 (26.7)	11 (7.8)	<0.001
Deaths, no. (%)	429	172 (40.1)	108 (37.5)	64 (45.4)	0.117
Lung transplants, no. (%)	338	13 (3.9)	8 (3.6)	5 (4.2)	0.784
Dx after 2015, no. (%)	429	237 (55.2)	153 (53.1)	84 (59.6)	0.207
Observation time, years (Q1–Q3)	429	3.3 (1.4**–**5.6)	3.6 (1.5–6.1)	2.9 (1.2–4.7)	0.027
1-, 3- and 5-year TFS (%)	429	93/78/64	93/80/69	93/73/53	0.006

Data are presented as no. (%), mean ± SD or median (Q1–Q3) as appropriate. SSc: systemic sclerosis; lcSSc: limited cutaneous systemic sclerosis; mRSS: modified Rodnan skin score; PAH: pulmonary arterial hypertension; ACA: anti-centromere antibody; FVC: forced vital capacity; DLCO: diffusing capacity of the lung for carbon monoxide; 6MWD: 6-min walk distance; WHO-FC: World Health Organization functional class; ILD: interstitial lung disease, limited extent; NT-proBNP: N-terminal brain natriuretic peptide; TAPSE/sPAP: tricuspid annular plane systolic excursion/systolic pulmonary artery pressure; mPAP: mean pulmonary arterial pressure; PAWP: pulmonary arterial wedge pressure; PVR: pulmonary vascular resistance; CI: cardiac index; Dx: diagnosis; TFS: transplant-free survival. *P*-values represent pairwise comparisons.

### Risk stratification at baseline

We identified 11 published PAH risk stratification tools in addition to the ESC/ERS three-strata model, which we applied as the reference tool (Supplementary Table S1, available at *Rheumatology* online). Based on their applicability and performance in the SSc-PAH cohort, the top three tools were selected for comparison against the reference tool (Supplementary Tables S2 and S3, available at *Rheumatology* online): (A) the ESC/ERS three-strata model (used as the reference tool), (B) the ESC/ERS four-strata model, (C) ‘SPAHR updated’ and (D) ‘REVEAL Lite 2’ (Fig. 1).

The number of patients fulfilling the inclusion criteria of each risk stratification tool varied. Patients who met the reference tool’s criteria had shorter SSc disease duration at PAH diagnosis, less pre-existing treatment, more upfront treatment and better TFS compared with those who did not (Supplementary Table S4, available at *Rheumatology* online). They were also more frequently diagnosed after 2015, when the ESC/ERS three-strata model and upfront combination therapy were introduced, potentially affecting tool fulfilment and outcomes (Supplementary Table S4, available at *Rheumatology* online). Due to overlapping populations across the four tools, statistical comparisons of patient characteristics were not feasible (Supplementary Table S5, available at *Rheumatology* online).

**Figure 2.**
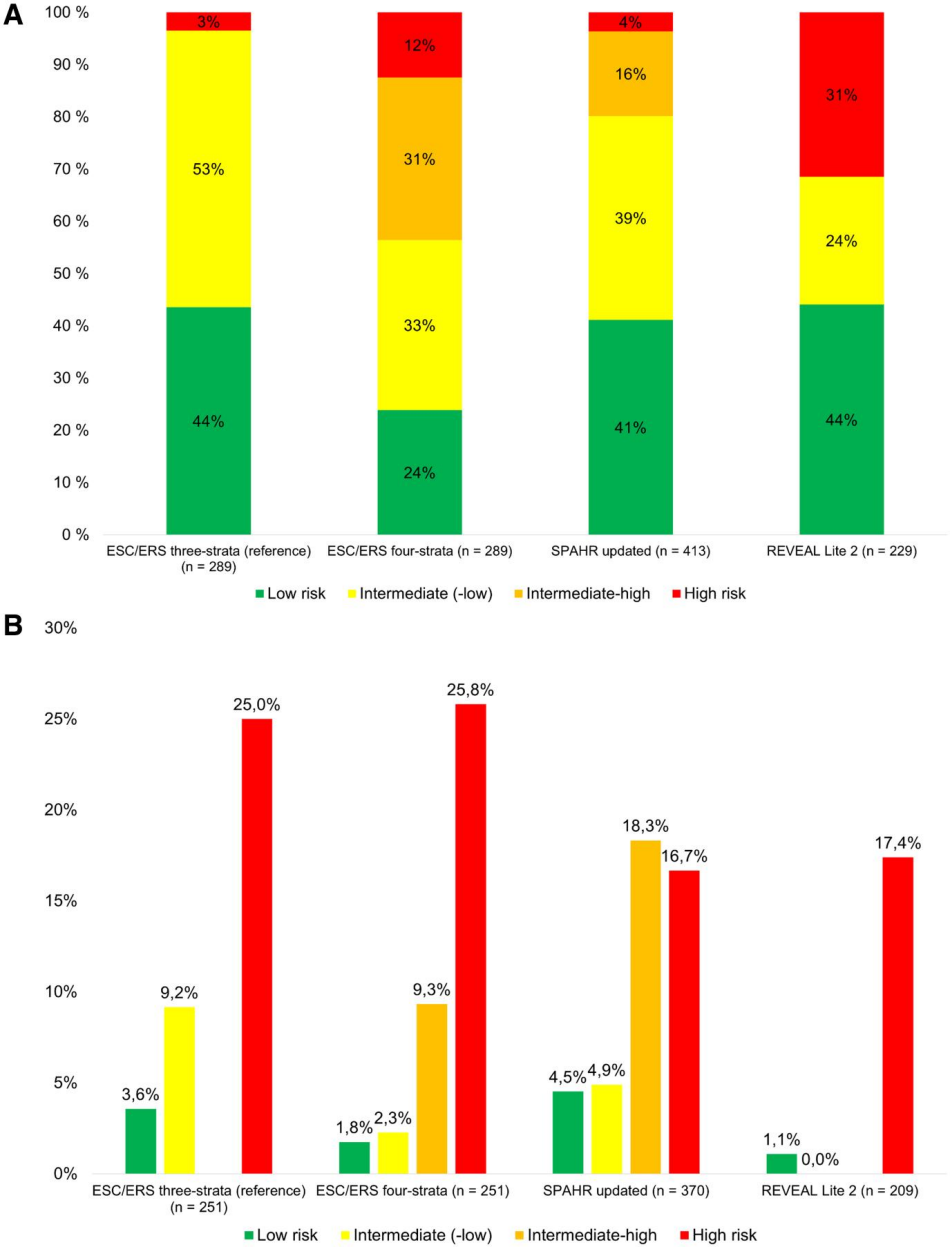
(A) Proportion of patients and (B) observed 1-year mortality across risk categories in the four risk stratification tools. ESC/ERS: European Society of Cardiology and European Respiratory Society; SPAHR: Swedish Pulmonary Arterial Hypertension Registry; REVEAL: Registry to Evaluate Early and Long-Term PAH Disease Management. The 1-year mortality rate was determined for patients who were either deceased or had at least a one-year observation period

Depending on the risk stratification tool applied, the distribution of risk groups varied (Fig. 2A). The ESC/ERS three-strata model classified 3% of patients as high-risk, while the majority fell into the intermediate- (53%) or low- (44%) risk categories. ‘SPAHR updated’ showed similar results but further subdivided the intermediate-risk group into two groups. The ESC/ERS four-strata model and ‘REVEAL Lite 2’ had a more uniform distribution of risk groups, with a higher proportion of patients classified as high-risk. No significant differences in the distribution of risk groups were observed between patients diagnosed before and after 2015 (Supplementary Table S6, available at *Rheumatology* online). Observed 1-year mortality rates by risk groups aligned with the expected rates as estimated by the guidelines for the ESC/ERS three- and four-strata models (Fig. 2B). Conversely, ‘SPAHR updated’ and ‘REVEAL Lite 2’ overestimated mortality for high-risk and intermediate- and high-risk groups, respectively.

**Figure 3.**
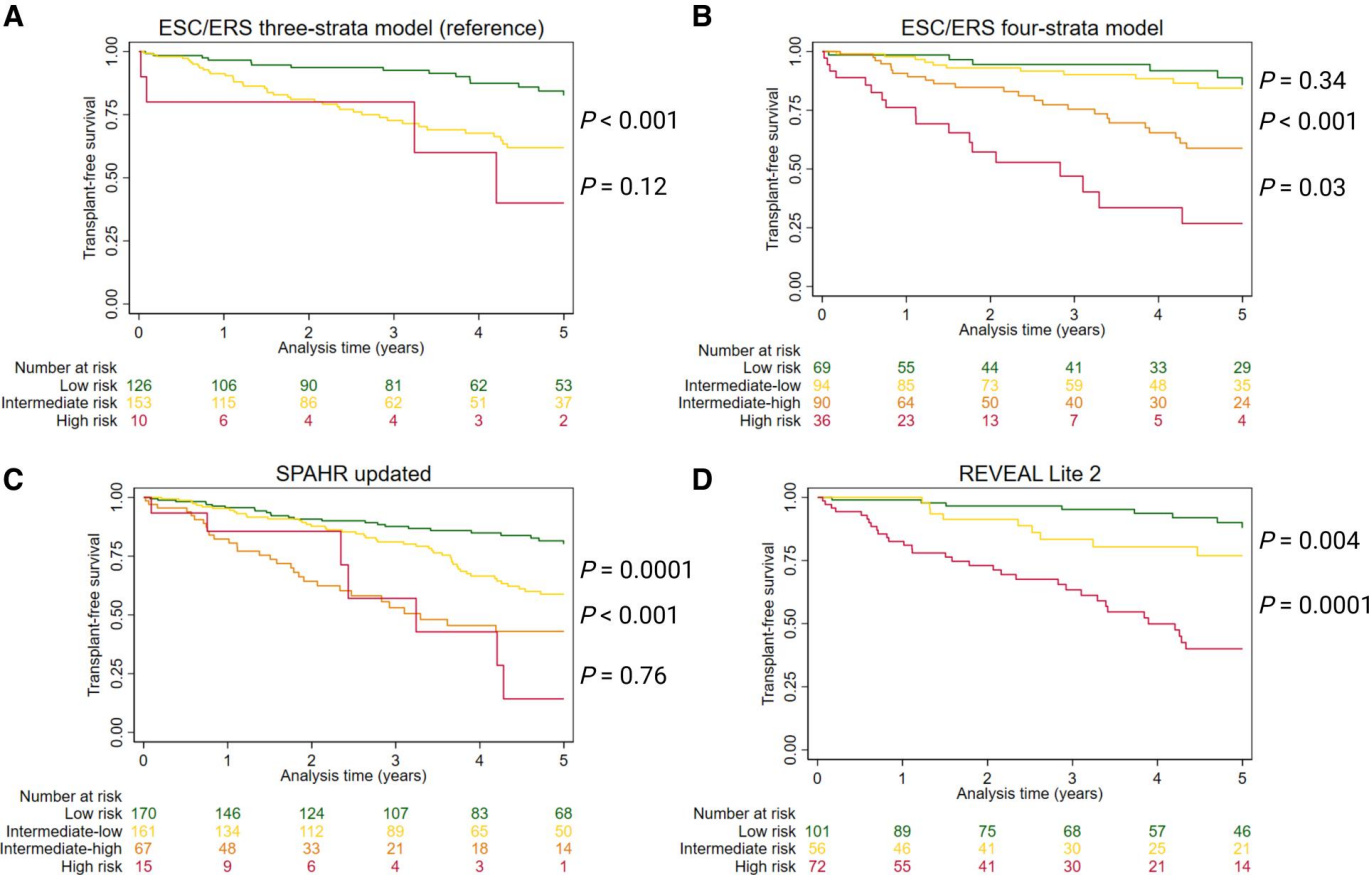
Transplant-free survival by risk groups in the four risk stratification tools. (A) ESC/ERS three-strata model, (B) ESC/ERS four-strata model, (C) ‘SPAHR updated’ and (D) ‘REVEAL Lite 2’. *P*-values for pairwise comparison of the risk groups using the log-rank test. ESC/ERS: European Society of Cardiology and European Respiratory Society; SPAHR: Swedish Pulmonary Arterial Hypertension Registry; REVEAL: Registry to Evaluate Early and Long-Term PAH Disease Management

TFS was differentiated across all risk strata using ‘REVEAL Lite 2’, while no significant differences were observed between the intermediate- and high-risk groups using the ESC/ERS three-strata model, or between the intermediate-high- and high-risk groups using ‘SPAHR updated’ (Fig. 3). The ESC/ERS four-strata model demonstrated significantly worse TFS in the intermediate-high-risk group compared with the intermediate-low-risk group, whose survival rates were comparable to the low-risk group.

**Figure 4.**
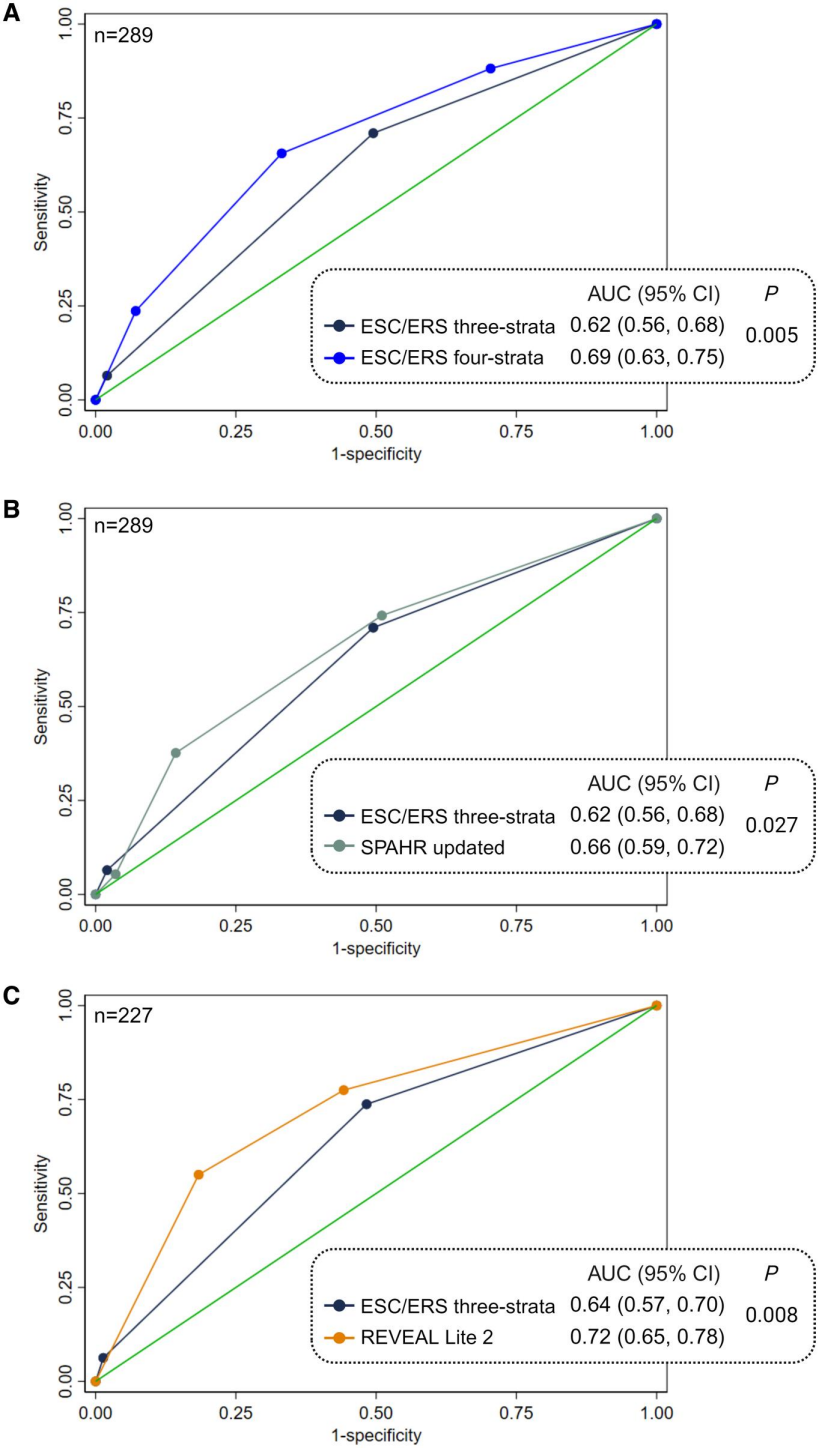
Performance of the risk stratification tools in predicting all-cause mortality compared with the ESC/ERS three-strata model (reference) in unadjusted analysis. (A**)** ESC/ERS four-strata model compared to the reference, (B) ‘SPAHR updated’ compared to the reference and (C) ‘REVEAL Lite 2’ compared to the reference. Predictive abilities were evaluated using area under the ROC curve (AUC) based on univariable Cox regression analysis, and performance was compared to the ESC/ERS three-strata model (reference). *P*-values represent the statistical significance of differences in predictive performance between the risk stratification tools. The graphs were generated using STATA and assembled in BioRender.com. ESC/ERS: European Society of Cardiology and European Respiratory Society; SPAHR: Swedish Pulmonary Arterial Hypertension Registry; REVEAL: Registry to Evaluate Early and Long-Term PAH Disease Management; AUC: area under the ROC curve

All the tools showed significantly greater ability to predict mortality compared with the ESC/ERS three-strata reference tool, as indicated by higher AUC values (Fig. 4). The ESC/ERS four-strata model and ‘SPAHR updated’, both of which stratify patients into four risk groups, showed a significantly higher mortality risk for the intermediate-high-risk groups compared with the intermediate-low-risk groups (Supplementary Table S7, available at *Rheumatology* online). The ESC/ERS three-strata model, the ESC/ERS four-strata model and ‘SPAHR updated’ did not significantly distinguish mortality risk between the intermediate- and high-risk groups, the low- and intermediate-low-risk groups, and the intermediate-high and high-risk groups, respectively (Supplementary Table S7, available at *Rheumatology* online).

Using the ESC/ERS four-strata model, WHO-FC, 6MWD and NT-proBNP were all significant predictors of intermediate-high risk classification compared to intermediate-low risk classification (Supplementary Fig. S4, available at *Rheumatology* online). NT-proBNP showed significantly higher predictive ability than WHO-FC (*P* = 0.007), while no significant difference was observed between WHO-FC and 6MWD (*P* = 0.55), or between 6MWD and NT-proBNP (*P* = 0.94).

### Impact of incorporating SSc-specific characteristics

**Figure 5.**
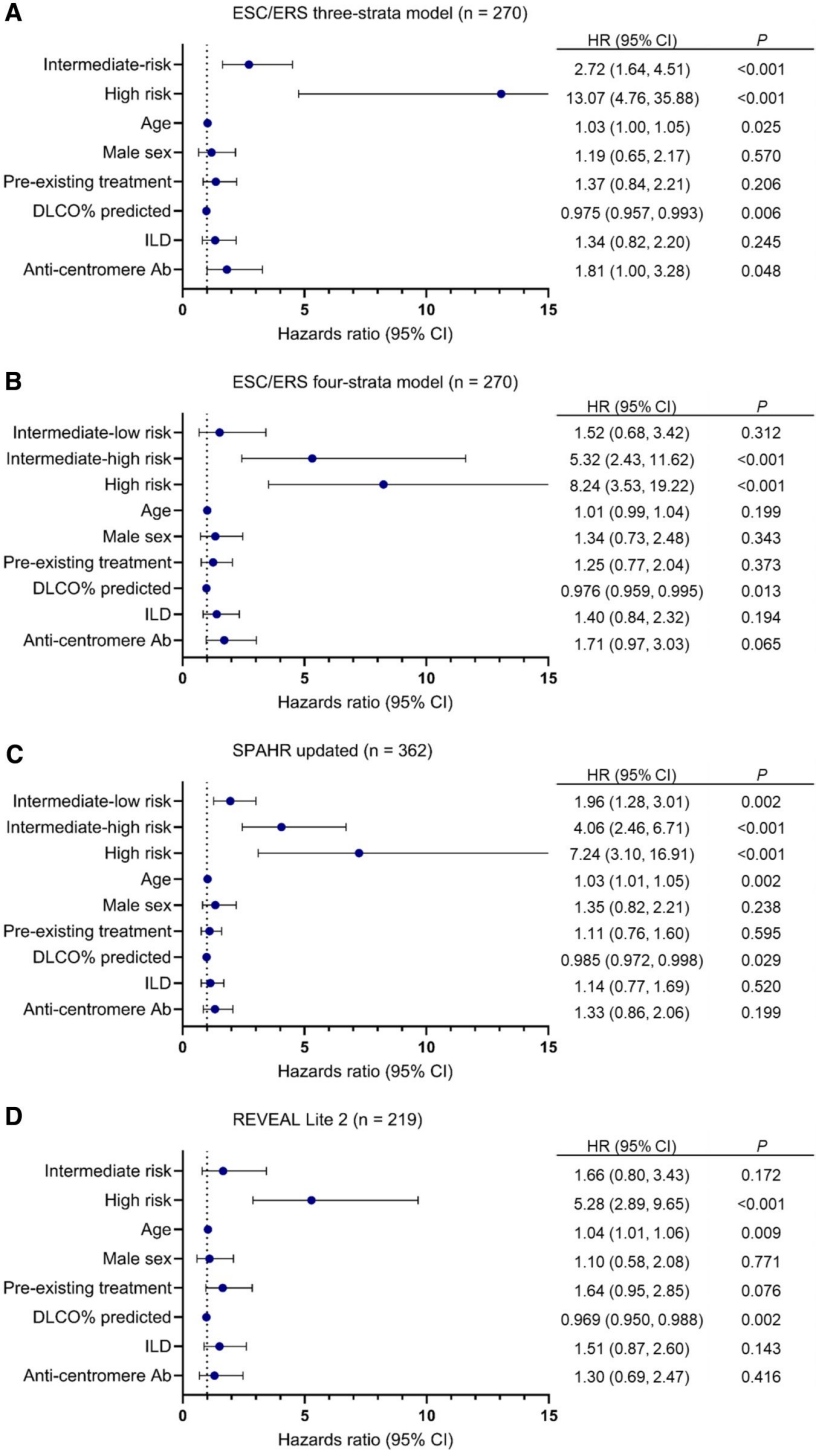
Impact of risk stratification tools on predicting all-cause mortality in multivariable analysis. (A) ESC/ERS three-strata model (reference), (B) ESC/ERS four-strata model, (C) ‘SPAHR updated’ and (D) ‘REVEAL Lite 2’. The multivariable models are adjusted for age, male sex, pre-existing vascular-targeted therapy, DLCO% predicted, ILD of limited extent and anti-centromere antibodies. Hazard ratios (HRs) and 95% CIs are shown for all variables. HRs for risk groups are referenced to the low-risk group. *P*-values represent the significance of the HRs obtained from multivariable Cox regression analyses. ESC/ERS: European Society of Cardiology and European Respiratory Society; SPAHR: Swedish Pulmonary Arterial Hypertension Registry; REVEAL: Registry to Evaluate Early and Long-Term PAH Disease Management; DLCO: diffusing capacity of the lung for carbon monoxide; ILD: interstitial lung disease, limited extent; Ab: antibodies; HR: hazard ratio

The final covariates for the multivariable models were selected based on availability, multicollinearity and model performance (Supplementary Table S8, available at *Rheumatology* online). The addition of age, male sex, pre-existing vascular-targeted therapies, DLCO% predicted, ILD of limited extent and anti-centromere antibodies did not significantly improve the predictive ability of the risk stratification tools (Supplementary Table S9, available at *Rheumatology* online). DLCO was the only predictor of mortality independent of the risk stratification tools across all the models (Fig. 5).

### Subgroup and sensitivity analyses

All analyses were also performed in treatment-naïve patients, yielding results comparable to the total cohort (Supplementary Figs S5–S7, Supplementary Tables S10–S12, available at *Rheumatology* online). In the subanalysis based on haemodynamic thresholds, patients with mPAP 21–24 mmHg and/or PVR 2–3 WU (*n*  = 118) demonstrated better risk profiles and TFS rates compared with those in the higher threshold group (Supplementary Tables S13–S15, Supplementary Fig. S8, available at *Rheumatology* online). There were too few events to perform Cox regression analyses confined within the lower threshold group. However, incorporating mPAP and PVR threshold groups, along with age, male sex, DLCO and pre-existing vascular-targeted therapies into the multivariable analysis, resulted in findings consistent with the primary analysis (Supplementary Fig. S9, available at *Rheumatology* online).

We also repeated the comparative analyses on the subset of patients meeting all four tool requirements and obtained similar findings (Supplementary Figs S10–S12, Supplementary Tables S16–S18, available at *Rheumatology* online). A direct comparison of the two top-performing univariable tools, the ESC/ERS four-strata model and ‘REVEAL Lite 2’, showed no significant differences in their ability to predict mortality (AUC 0.73 [95% CI 0.66, 0.79] *vs* AUC 0.72 [95% CI 0.65, 0.78], *P* = 0.646).

The sensitivity analysis with multiple imputations resulted in no notable variations in the results (Supplementary Fig. S13, Supplementary Table S19, available at *Rheumatology* online).

## Discussion

In this study, we aimed to identify the most accurate approach for risk stratification at the time of SSc-PAH diagnosis, comparing several tools to the ESC/ERS three-strata model, the currently recommended tool for baseline risk assessment. We also explored whether incorporating SSc-specific characteristics could enhance the predictive accuracy of these tools.

In our cohort of newly diagnosed SSc-PAH patients, according to the 2022 haemodynamic definition, we found that the currently recommended ESC/ERS three-strata model had a lower ability to predict mortality than the ESC/ERS four-strata model, ‘SPAHR updated’ and ‘REVEAL Lite 2’. The ESC/ERS four-strata model effectively divided ‘intermediate-risk’ patients into two groups with significantly different long-term survival rates and includes the most clinically accessible risk parameters. Although incorporating SSc-specific characteristics did not significantly improve predictive ability, low DLCO was identified as an independent predictor of mortality.

Previous studies on risk stratification in SSc-PAH have often been limited to single-centre studies, subgroup analyses or focused on treatment-naïve patients using the 2015 haemodynamic criteria [12, 21–28, 41–44]. Our study uniquely evaluates, to our knowledge, all published risk stratification tools within a single comparator study, offering a comprehensive assessment of these tools in a multicentre, international cohort of SSc-PAH patients from the EUSTAR database. Importantly, our cohort includes patients with pre-existing therapies for vascular symptoms, such as Raynaud phenomenon and DUs, as well as those fulfilling the 2022 haemodynamic definition of PAH.

We ranked the 11 identified PAH risk stratification tools by their applicability and performance in the SSc-PAH cohort, comparing the top three to the ESC/ERS three-strata model as a reference [19]. All three tools demonstrated a significantly greater ability to predict mortality than the ESC/ERS three-strata model. Notably, the ESC/ERS three-strata model did not significantly differentiate mortality risk between intermediate- and high-risk patients. This has important therapeutic implications, especially considering the different upfront treatment recommendations, including upfront triple therapy for the high-risk group, as outlined in the 2022 guidelines and further reinforced in the recent update from the seventh WSPH [19, 45].

Furthermore, the ESC/ERS three-strata model classified most patients as intermediate risk, with only 3% as high-risk. Previous studies have shown that subdividing the intermediate-risk group improves outcome differentiation and increases sensitivity to change during follow-up [21–23, 41]. In this study, both the ESC/ERS four-strata model and ‘SPAHR updated’ successfully divided intermediate-risk patients into subgroups with significantly different long-term survival rates. However, ‘SPAHR updated’ did not distinguish mortality risk between intermediate-high and high-risk patients, and only 4% of patients were classified as high-risk, with a lower than expected 1-year mortality rate. This suggests that the tool may overestimate mortality for the high-risk group.

The ESC/ERS four-strata model and ‘REVEAL Lite 2’ demonstrated a uniform distribution of risk groups, with a higher proportion stratified as high-risk. The tools demonstrated significant discrimination of mortality risk across risk strata, except between the low- and intermediate-low-risk groups in the ESC/ERS four-strata model. However, since the primary goal of baseline risk assessment is to identify high-risk patients for upfront triple therapy, this distinction is of lesser clinical importance [19–21]. There was no significant difference in mortality prediction in direct comparison between the ESC/ERS four-strata model and ‘REVEAL Lite 2’. However, while the ESC/ERS four-strata model correctly aligned 1-year mortality rates with expected values, ‘REVEAL Lite 2’ overestimated mortality for intermediate- and high-risk patients in this cohort, leading to less precise risk stratification [19, 21, 41]. In addition to its predictive ability, effective subdivision of the intermediate-risk group and accurate estimation of 1-year mortality, the ESC/ERS four-strata model is practical for clinical use, relying on three easily accessible parameters (WHO-FC, 6MWD and BNP/NT-proBNP), which have previously shown the greatest prognostic value in PAH [24, 26–29]. Notably, these three risk parameters were all significant predictors when distinguishing between intermediate-low and intermediate-high risk groups, with NT-proBNP being the strongest.

The current treatment algorithm differentiates between low- and intermediate-risk *vs* high-risk patients [19, 45]. However, our study shows that intermediate-high-risk patients had a significantly worse prognosis compared to lower risk groups, suggesting that this subgroup may require a different management approach. Future randomized controlled trials are needed to determine the optimal treatment strategies for these patients. In addition to guiding treatment decisions, precise risk stratification is crucial for providing prognostic information and monitoring changes over time. While identifying intermediate-high-risk patients may not lead to immediate treatment changes, it enables the opportunity for closer surveillance and potentially earlier intervention. Given the poor prognosis in this group, we propose heightened awareness, including guideline-aligned treatment and careful monitoring.

Our study also assessed whether incorporating SSc-specific factors could improve predictive accuracy in the tools. Although including these factors did not significantly enhance the predictive ability of the tools, a low DLCO was an independent predictor of mortality. Numerous studies have shown that patients with SSc-PAH have lower DLCO than those with IPAH [4, 5, 46] and that a lower DLCO is associated with a poorer outcomes [33–36]. However, it remains unclear whether DLCO can improve following PAH-specific treatment, which is crucial when considering its potential role in risk stratification, particularly for follow-up assessments where sensitivity to change is essential. Some studies suggest that patients with very low DLCO may respond less effectively to therapy and that treatment may further impair gas exchange in these patients [47, 48]. While adding DLCO to risk stratification may not directly change treatment strategies, its prognostic role underscores its importance in a comprehensive risk evaluation. In a broader context, systemic organ involvement is well-documented to impact treatment response and disease outcomes in SSc patients [6, 9, 10, 32–40]. Therefore, even though SSc-specific factors did not improve the predictive accuracy of the tools *per se*, SSc-related organ involvement and comorbidities should still be considered in the overall clinical assessment for prognostic evaluation and treatment decisions, alongside other individual factors, as recommended by the guidelines [19, 20, 45].

Previous studies largely focused on treatment-naïve patients when evaluating baseline risk stratification, but many SSc patients in clinical practice are already receiving therapies, such as ERAs, PDE-5is and PPAs, to manage vascular symptoms like Raynaud phenomenon and DUs. Our study reflects real-world conditions by including both treatment-naïve patients and those with pre-existing vascular-targeted therapies. Importantly, the subanalysis of treatment-naïve patients showed comparable performance in predicting mortality to that of the overall cohort, supporting the robustness of risk stratification at the time of SSc-PAH diagnosis, regardless of prior treatment status.

The management approach for patients with milder haemodynamic impairment (mPAP of 21–24 mmHg or PVR of 2–3 WU) remains uncertain, with close monitoring and individualized treatment decisions recommended [19, 45]. Therefore, evidence on the efficacy of risk stratification in these patients is crucial. In our cohort, patients with lower mPAP and PVR thresholds demonstrated better risk profiles and prognoses, though some were still classified at higher risk despite their milder haemodynamic burden. Tools that do not incorporate haemodynamic variables may be influenced by other factors, such as heart failure from non-PAH causes, lung disease or musculoskeletal limitations [20]. In our study, intermediate-high and high-risk patients with milder haemodynamic impairment had no major differences in SSc-related organ manifestations compared with lower-risk groups. However, unmeasured factors or subtle clinical features may contribute to the elevated risk in these patients, highlighting the need for an individualized approach. We found that risk stratification was effective independent of haemodyamic thresholds, supporting its utility even in patients with milder haemodynamic impairment.

As with all registry analyses, our study has limitations, including missing data, lack of standardized follow-up and the inclusion of patients diagnosed over an extended period with evolving screening recommendations, diagnostic criteria and management strategies, which increases population heterogeneity. A potential limitation is that the results may not fully apply to patients outside expert centres. However, since the 2022 ESC/ERS guidelines recommend that all SSc-PAH patients be managed in expert centres, this should not significantly affect generalizability. Although patients diagnosed after 2015 more frequently met the risk stratification criteria, there were no significant differences in risk group distribution or survival. This suggests that the time of diagnosis did not significantly impact the effectiveness of risk stratification. The retrospective application of the 2022 haemodynamic definition partly explains why many patients did not receive upfront therapy. While pre-existing vascular-targeted therapies likely influenced upfront treatment decisions, including pretreated patients makes our results more reflective of clinical practice. The accuracy of risk stratification remained comparable between treatment-naïve and pretreated patients. Although missing data are inherent to registry studies, subanalysis of patients meeting all risk stratification criteria and sensitivity analyses using multiple imputation did not substantially alter our findings. Unfortunately, data required to assess risk stratification at follow-up were not available.

In conclusion, considering the overall ability to predict mortality, risk segregation capabilities and clinical applicability, this study provides a rationale for using the simplified ESC/ERS four-strata model in SSc-PAH at the time of diagnosis as an alternative to the comprehensive ESC/ERS three-strata model. Risk stratification was accurate in SSc-PAH patients, regardless of pre-existing vascular-targeted therapies or haemodynamic thresholds. We also propose considering DLCO as a prognostic marker in baseline risk assessment for SSc-PAH patients, alongside other individual factors recommended by the guidelines [19, 20]. Further research is needed to optimize the management of intermediate-high-risk patients and to explore temporal changes in DLCO in SSc-PAH patients.

## Supplementary Material

keaf053_Supplementary_Data

## Data Availability

The data underlying this article will be shared on reasonable request to the corresponding author.

## AI statement

During the preparation of this work, the authors used QuillBot (https://quillbot.com/) and OpenAI’s ChatGPT (GPT-4; https://openai.com/chatgpt/) for assistance in refining the manuscript, including grammar checking, optimizing phrasing, and improving conciseness. After using these tools, the authors reviewed and edited the content as needed and take full responsibility for the content of the publication. Some figures in this manuscript were created using BioRender.com and are credited accordingly in the figure legends.
