# Supplementary material for: A comparative analysis of risk stratification tools in SSc-associated pulmonary arterial hypertension: a EUSTAR analysis
Source: Rheumatology (Oxford). 2025 Jan 29;64(6):3643–56. doi: 10.1093/rheumatology/keaf053 (PMC12107028; doi:10.1093/rheumatology/keaf053)
Supplement: keaf053_Supplementary_Data [file keaf053_supplementary_data.zip › keaf053_Supplementary_Data/rhe-24-2469-File008.pdf]

Appendix A

List of EUSTAR collaborators:

- Florence (Italy), Serena Guiducci (001);
- Bari (Italy), Florenzo Iannone (004);
- Cluj-Napoca (Romania), Simona Rednic (016);
- Paris (France), Yannick Allanore (017);
- Pavia (Italy), Carlomaurizio Montecucco (019);
- Pecs (Hungary), Gábor Kumánovics (025);
- Geneva (Switzerland), Michele Iudici (028);
- Ancona (Italy), Gianluca Moroncini (034);
- Lund (Sweden), Kristofer Andréasson (040);
- Verona (Italy), Luca Idolazzi (050);
- Tübingen (Germany), Jörg Henes (056);
- Stuttgart (Germany), Johannes Pflugfelder (058);
- Coimbra (Portugal), José António Pereira da Silva (068);
- Salford (United Kingdom), Michael Hughes (080);
- Rome (Italy), Valeria Riccieri (094);
- Bucharest (Romania), Andra Balanescu (096);
- Bucharest (Romania), Ana Maria Gheorghiu (100);
- Erlangen (Germany), Christina Bergmann (106);
- Foggia (Italy), Francesco Paolo Cantatore (115);
- Leuven (Belgium), Ellen De Langhe (126);
- Zagreb (Croatia), Branimir Ani (128);
- Curitiba (Brazil), Carolina de Souza Müller (135);
- Hamilton (New Zealand), Kamal Solanki (148);
- Rome (Italy), Edoardo Rosato (158);
- Bern (Switzerland), Britta Maurer (164);
- New Orleans (USA), Lesley Ann Saketkoo (177);
- Bergamo (Italy), Massimiliano Limonta (182);
- New Brunswick (USA), Vivien M. Hsu (188);
- Stanford (USA), Lorinda S Chung (191);
- kfar-saba (Israel), Yair Levy (201);
- Athens (Greece), Petros Sfikakis (213);
- Amadora (Portugal), Susana Oliveira (223);
- Tokyo (Japan), Masataka Kuwana (225).
