# Supplementary material for: A comparative analysis of risk stratification tools in SSc-associated pulmonary arterial hypertension: a EUSTAR analysis
Source: Rheumatology (Oxford). 2025 Jan 29;64(6):3643–56. doi: 10.1093/rheumatology/keaf053 (PMC12107028; doi:10.1093/rheumatology/keaf053)
Supplement: keaf053_Supplementary_Data [file keaf053_supplementary_data.zip › keaf053_Supplementary_Data/rhe-24-2469-File009.docx]

**Supplementary material**

**Supplementary Figure S1:** Strengthening the Reporting of Observational Studies in Epidemiology diagram showing patient eligibility for analysis.


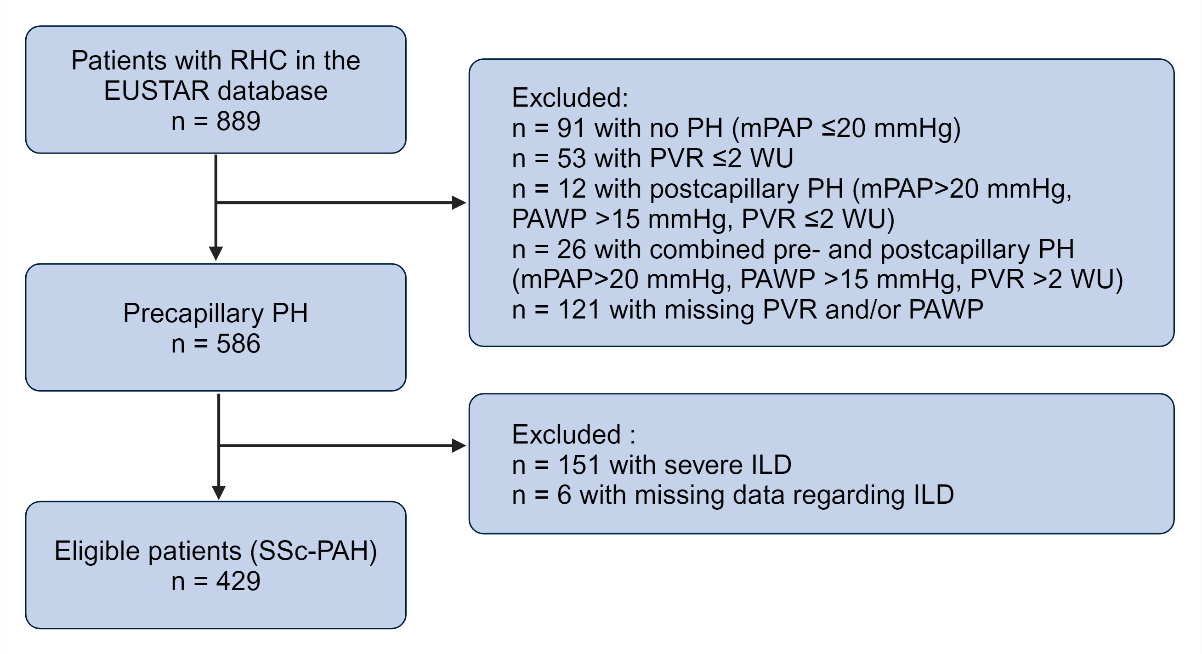


RHC: right heart catheterization; EUSTAR: European Scleroderma Trials and Research group; PH: pulmonary hypertension; SSc-PAH: systemic sclerosis-associated pulmonary arterial hypertension; mPAP: mean pulmonary arterial pressure; PVR: pulmonary vascular resistance; PAWP: pulmonary artery wedge pressure; ILD: interstitial lung disease. Created in BioRender.com.

**Supplementary Figure S2:** Transplant-free survival in patients with pre-existing vascular-targeted therapies vs. treatment-naïve patients.

**
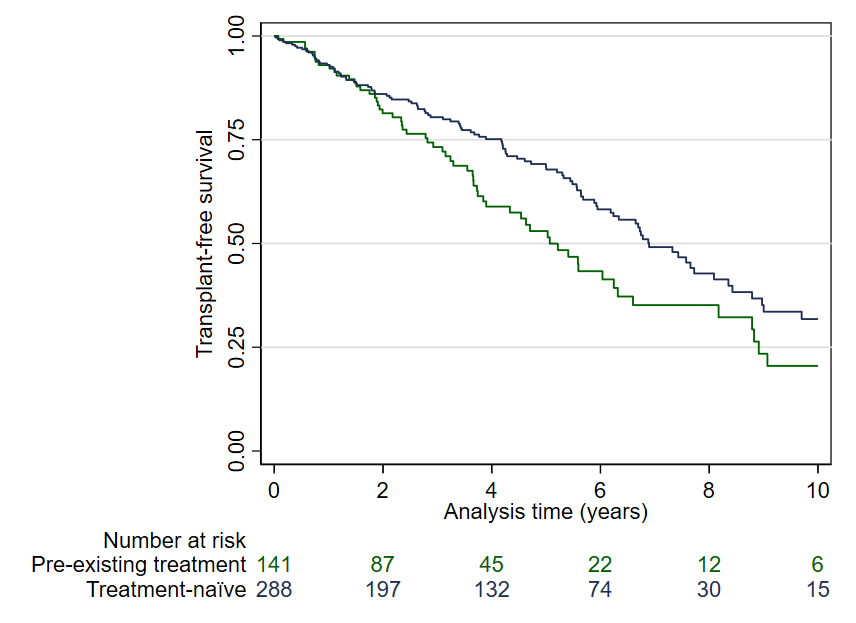
**

Log-rank p-values represents the statistical comparison of survival curves between the groups (log-rank p=0.006).

**Supplementary Figure S3:** Transplant-free survival in patients diagnosed with SSc-PAH before 2015 vs. after 2015.


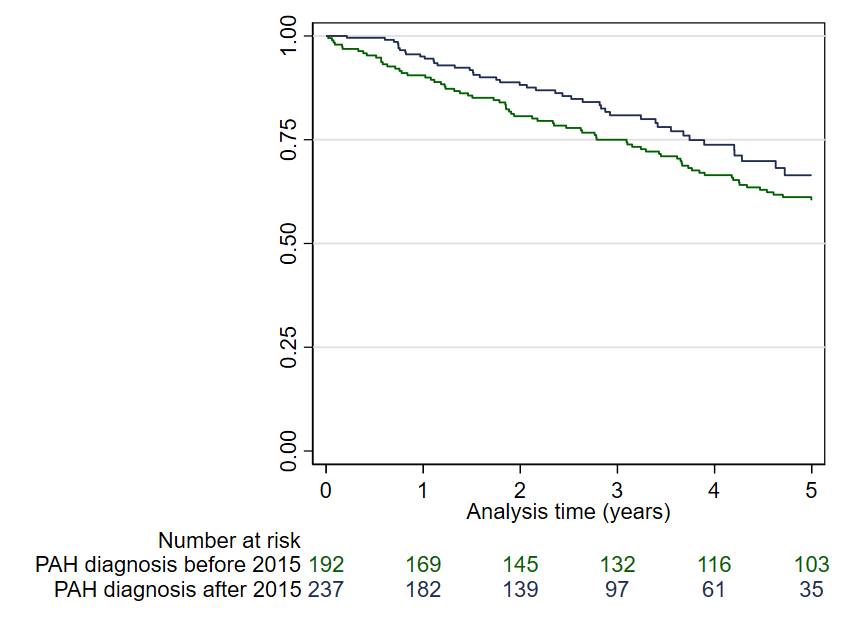


Log-rank p-values represents the statistical comparison of survival curves between the groups (log-rank p=0.121).

**Supplementary Table S1:** Description of the published risk stratification tools.

| 1. The ESC/ERS three-strata model (reference) (Humbert et al., 2022, Eur Heart J) | Stratifies patients into three risk groups based on up to 18 parameters, with at least World Health Organization functional class (WHO-FC) and/or 6-minute walking distance (6MWD), and N-terminal pro-brain natriuretic peptide (NT-proBNP) or BNP available. Each parameter was graded 1 to 3 representing low to high risk based on the cut-off values from the Guidelines, with the mean defining the risk category; low risk: mean score <1.50; intermediate risk: 1.50-2.49; and high risk: ≥2.50 |
| --- | --- |
| 2. The simplified ESC/ERS four-strata model (Hoeper et al., 2022, Eur Respir J) | Stratifies patients into four risk groups based on refined cut-off levels of at least WHO-FC and/or 6MWD and NT-proBNP/BNP with an estimated 1-year mortality of 0–3%, 2–7%, 9–19%, and >20%, respectively. Each parameter was graded 1 to 4 representing low to high risk, with the mean defining the risk category; low risk: mean score <1.50; intermediate-low risk: 1.50-2.49; intermediate-high risk: 2.50-3.49; and high risk: ≥3.50. |
| 3. SPAHR updated  (Ahmed et al., 2023, Eur Heart J Open) | Stratifies patients into four risk groups based on up to 11 variables with cut-off values from the risk table in the 2022 ESC/ERS Guidelines, with at least 3 variables available of; clinical signs of right heart failure, progression of symptoms, syncope, WHO-FC, 6MWD, NT-proBNP/BNP, right atrial area, pericardial effusion, right atrial pressure (RAP), cardiac index (CI), and mixed-venous oxygen saturation (SvO2). The parameters were graded as described in (1), and divided into four risk groups based on the mean score: low risk: mean score <1.50; intermediate-low risk: 1.5-1.99; and intermediate-high risk: 2.0-2.49; and high risk: ≥2.50 |
| 4. REVEAL Lite 2 (Benza et al., 2021, Chest) | Stratifies patients into three risk groups based on six weighted variables; WHO-FC, 6MWD, NT-pro-BNP/BNP, systolic blood pressure (SBP), heart rate (HR) and estimated glomerular filtration rate (eGFR), with at least 3 variables available, whereof at least 2 of the most predictive (WHO-FC, 6MWD and/or NT-proBNP/BNP). Each patient begins with a score of 6, and has 0-2 points added or subtracted according to the cut-offs for each parameter; low risk: risk score ≤5; intermediate risk: 6-7; and high risk: ≥8 |
| 5. REVEAL 2.0 (Benza et al., 2019, Chest) | Stratifies patients into three risk groups by inclusion of 13 weighted modifiable and nonmodifiable variables; PAH subgroup, male age >60 years, eGFR, WHO-FC, SBP, HR, all-cause hospitalizations ≤6 months, 6MWD, NT-proBNP/BNP, pericardial effusion, DLCO% predicted, RAP, and PVR. Each patient begins with a score of 6 and has 1 or 2 points added or subtracted for each parameter. A minimum of 7 variables are required to generate a score. Low risk: risk score ≤ 6; intermediate risk: risk score 7 – 8; high risk: risk score ≥ 9 |
| 6. The 2022 ESC/ERS model in four groups (Kylhammar et al., 2021, ERJ Open Res) | Equals number 1, but the intermediate-risk group was divided into two groups based on the mean score; intermediate-low risk: risk score 1.5-1.99; and intermediate-high risk: 2.0-2.49 |
| 7.SPAHR/COMPERA 3-strata (Kylhammar et al., 2018, Eur Heart J, Hoeper et al., 2017, Eur Respir J) | Stratifies patients into three groups based on up to 8 variables from the risk table in the guidelines, with at least 3 variables available of; WHO-FC, 6MWD, NT-proBNP, right atrial area, pericardial effusion, RAP, CI and/or SvO2. The cut-off values were adjusted to the 2022 ESC/ERS guidelines. Each parameter was graded from 1-3 representing low-high risk. The mean score defined three risk groups as described in (1) |
| 8. SPAHR 4-strata (Kylhammar et al., 2021, ERJ Open Res) | Equals the SPAHR/COMPERA 3-strata strategy, but the intermediate risk group is further divided into an intermediate-low (risk score 1.5-1.99) and an intermediate-high (risk score 2.0-2.49) risk groups based on the mean score |
| 9. SPAHR - Proportion of low-risk parameters (Kylhammar et al., 2021, ERJ Open Res) | Stratifies patients into four groups based on the proportion of the low-risk parameters described in (7); 0-24% = high risk; 25-49% = intermediate-high risk; 50-74% = intermediate-low risk; 75-100% = low risk |
| 10. FPHR - Number of low-risk criteria (Boucly et al., 2017, Eur Respir J) | Stratifies patients according to the number of 4 low-risk criteria from the risk table in the guidelines, with all variables available of; WHO-FC, 6MWD, RAP, CI; resulting in 5 groups; 0, 1, 2, 3 or 4 low-risk criteria |
| 11. FPHR - Number of three non-invasive low-risk criteria  (Boucly et al., 2017, Eur Respir J) | Stratifies patients according to the number of 3 non-invasive low-risk criteria from the risk table in the guidelines, with all variables available of; WHO-FC, 6MWD and BNP/NT-proBNP; resulting in 4 groups; 0, 1, 2 or 3 low-risk criteria |
| 12. Bologna  (Dardi et al., 2021, Open Heart) | Stratifies patients into three groups based on WHO-FC, 6MWD, NT-proBNP/BNP or RAP and CI or SvO2 with cut off values based on the 2015 ESC/ERS guidelines, with low risk defined as ≥3 low-risk criteria and no high-risk criteria, high risk defined as ≥2 high-risk criteria including CI or SvO2, and intermediate-risk defined as low or high risk not fulfilled |

SPAHR: ESC/ERS: European Society of Cardiology and European Respiratory Society; SPAHR: Swedish Pulmonary Arterial Hypertension Registry; COMPERA: Comparative, Prospective Registry of Newly Initiated Therapies for PH; REVEAL: Registry to Evaluate Early and Long-Term PAH Disease Management; FPHR: French Pulmonary Hypertension Registry.

**Supplementary Table S2:** Proportion of patients fulfilling the risk stratification tools and predictive ability for all-cause mortality using univariable Cox regression with area under the ROC curve (AUC) in the total SSc-PAH cohort (n = 429).

|  | No. (%) of patients fulfilling the criteria of the tool | AUC (95% CI) |
| --- | --- | --- |
| ESC/ERS three-strata model (reference)^a^ | 289 (67) | 0.62 (0.56, 0.68) |
| ESC/ERS four-strata model ^a^ | 289 (67) | 0.69 (0.63, 0.75) |
| SPAHR updated ^a^ | 413 (96) | 0.65 (0.60, 0.70) |
| REVEAL Lite 2 ^a^ | 229 (53) | 0.71 (0.64, 0.78 |
| REVEAL 2.0 | 334 (78) | 0.69 (0.63, 0.74) |
| ESC/ERS in 4-groups | 289 (67) | 0.64 (0.58, 0.71) |
| SPAHR/COMPERA 3-strata | 412 (96) | 0.62 (0.58, 0.67) |
| SPAHR 4-strata | 412 (96) | 0.64 (0.59, 0.69) |
| SPAHR – proportion low risk | 412 (96) | 0.64 (0.58, 0.69) |
| FPHR – nr of low-risk criteria (invasive) | 75 (17) | 0.79 (0.70, 0.89) |
| FPHR – nr of low-risk criteria (noninvasive) | 202 (47) | 0.70 (0.63, 0.77) |
| Bologna – 3 groups | 190 (44) | 0.66 (0.59, 0.72) |

ESC/ERS: European Society of Cardiology and European Respiratory Society; SPAHR: Swedish Pulmonary Arterial Hypertension Registry; COMPERA: Comparative, Prospective Registry of Newly Initiated Therapies for PH; REVEAL: Registry to Evaluate Early and Long-Term PAH Disease Management; FPHR: French Pulmonary Hypertension Registry; AUC: area under the ROC curve; CI: confidence interval. ^a^The tools that were selected for the primary analysis.

**Supplementary Table S3:** Proportion of patients with available data for risk variables used in risk assessment in patients meeting the criteria of the ESC/ERS three- and four-strata models, “SPAHR updated”, and “REVEAL Lite 2”, respectively.

|  | ESC/ERS three-strata model  (n = 289) | ESC/ERS four-strata model  (n = 289) | SPAHR updated  (n = 413) | REVEAL Lite 2 (n = 229) |
| --- | --- | --- | --- | --- |
| Signs of RHF, no. (%) | 104 (36) | N/A | 106 (26) | N/A |
| Progression of symptoms, no. (%) | 103 (36) | N/A | 105 (25) | N/A |
| Syncope, no. (%) | 103 (36) | N/A | 105 (25) | N/A |
| WHO-FC, no. (%) | 282 (98) | 282 (98) | 405 (98) | 228 (100) |
| 6MWD, no. (%) | 209 (72) | 209 (72) | 306 (74) | 204 (89) |
| Peak VO2, no. (%) | 5 (2) | N/A | N/A | N/A |
| VE/VCO2 slope, no. (%) | 0 | N/A | N/A | N/A |
| BNP or NT-proBNP, no. (%) | 289 (100) | 289 (100) | 290 (70) | 227 (99) |
| RA area, no. (%) | 99 (34) | N/A | 110 (27) | N/A |
| TAPSE/sPAP, no. (%) | 138 (48) | N/A | N/A | N/A |
| Pericardial effusion, no. (%) | 267 (92) | N/A | 378 (92) | N/A |
| cMRI RVEF, no. (%) | 21 (7) | N/A | N/A | N/A |
| cMRI SVI, no. (%) | 22 (8) | N/A | N/A | N/A |
| cMRI RVESVI, no. (%) | 19 (7) | N/A | N/A | N/A |
| RHC RAP, no. (%) | 99 (34) | N/A | 100 (24) | N/A |
| RHC CI, no. (%) | 272 (94) | N/A | 385 (93) | N/A |
| RHC SVI, no. (%) | 101 (35) | N/A | N/A | N/A |
| RHC SvO2, no. (%) | 99 (34) | N/A | 100 (24) | N/A |
| Systolic blood pressure, no. (%) | N/A | N/A | N/A | 105 (46) |
| Heart rate, no. (%) | N/A | N/A | N/A | 103 (45) |
| eGFR, no. (%) | N/A | N/A | N/A | 105 (46) |

ESC/ERS: European Society of Cardiology and European Respiratory Society; SPAHR: Swedish Pulmonary Arterial Hypertension Registry; REVEAL: Registry to Evaluate Early and Long-Term PAH Disease Management; RHF: right heart failure; WHO-FC: World Health Organization functional class, 6MWD: 6-minute walk distance; CPET: cardiopulmonary exercise testing, VO2: oxygen uptake; VE/VCO2: ventilatory equivalents for carbon dioxide; BNP: brain natriuretic peptide; NT-proBNP: N-terminal pro-brain natriuretic peptide; RA: right atrium; TAPSE: tricuspid annular plane systolic excursion; sPAP: systolic pulmonary arterial pressure; cMRI: cardiac magnetic resonance imaging; RVEF: right ventricular ejection fraction; SVI: stroke volume index; RVESVI: right ventricular end-systolic volume index; RAP: right atrial pressure; CI: cardiac index; SvO2: mixed venous oxygen saturation; eGFR: estimated glomerular filtration rate.

The risk variables presented are those included in each respective tool. The number of patients and the number of risk variables vary across the tools due to different criteria and parameters used in each model. Percentages represent the proportion of patients with available data for each risk variable within the respective tool. Cells marked 'N/A' indicate that the corresponding risk variable is not part of the respective tool.

**Supplementary Table S4:** Comparison of characteristics of patients fulfilling the ESC/ERS three-strata model (reference tool) vs. patients who did not fulfil the criteria for the reference tool.

|  | No. | All SSc-PAH  (n = 429) | No. | Fulfill criteria  (n = 289) | No. | Not fulfill criteria (n = 140) | *P* |
| --- | --- | --- | --- | --- | --- | --- | --- |
| Age, years | 429 | 65 ± 11 | 289 | 65 ± 11 | 140 | 66 ± 10 | 0.273 |
| Male sex, no. (%) | 429 | 60 (14.0) | 289 | 45 (15.6) | 140 | 15 (10.7) | 0.174 |
| **SSc characteristics** |  |  |  |  |  |  |  |
| SSc duration, years | 406 | 9.8 (3.7-16.5) | 268 | 8.7 (3.1-15.5) | 138 | 12.5 (4.2-18.0) | **0.019** |
| lcSSc, no. (%) | 420 | 342 (81.4) | 285 | 233 (81.8) | 135 | 109 (80.7) | 0.803 |
| mRSS, mean | 361 | 4.4 ± 6.2 | 259 | 5.6 ± 6.6 | 102 | 1.4 ± 3.3 | **<0.001** |
| ACA, no. (%) | 427 | 273 (64.0) | 289 | 177 (61.3) | 138 | 96 (69.6) | 0.094 |
| Digital ulcers, no. (%) | 423 | 170 (40.2) | 286 | 120 (42.0) | 137 | 50 (36.5) | 0.284 |
| Teleangiectasia, no. (%) | 417 | 352 (84.4) | 283 | 243 (85.9) | 134 | 109 (81.3) | 0.234 |
|  |  |  |  |  |  |  |  |
| Joint synovitis, no. (%) | 387 | 57 (14.7) | 259 | 43 (16.6) | 128 | 14 (10.9) | 0.139 |
| Muscle weakness, no. (%) | 359 | 60 (16.7) | 239 | 37 (15.5) | 120 | 23 (19.2) | 0.377 |
| Renal crisis, no. (%) | 407 | 16 (3.9) | 285 | 13 (4.6) | 122 | 3 (2.5) | 0.317 |
| **Lung characteristics** |  |  |  |  |  |  |  |
| FVC, % predicted | 408 | 91.3 ± 21.1 | 284 | 89.7 ± 21.2 | 124 | 94.8 ± 20.6 | **0.025** |
| DLCO, % predicted | 382 | 43 (33-52) | 271 | 43 (33-52) | 111 | 44 (33-53) | 0.402 |
| 6MWD, m | 306 | 341 ± 127 | 209 | 353 ± 131 | 97 | 317 ± 117 | **0.020** |
| WHO-FC III and IV, no. (%) | 418 | 211 (50.5) | 282 | 145 (51.4) | 136 | 66 (48.5) | 0.580 |
| ILD, no. (%) | 429 | 187 (43.6) | 289 | 146 (50.5) | 140 | 41 (29.3) | **<0.001** |
| **Heart characteristics** |  |  |  |  |  |  |  |
| NT-proBNP, ng/L | 260 | 568 (203-1495) | 256 | 568 (202-1495) | 4 | 621 (367-1463) | 0.743 |
| Right atrial area, cm^2^ | 111 | 17.6 (14.9-22.0) | 99 | 18.0 (15.0-22.0) | 12 | 16.2 (14.1-20.0) | 0.401 |
| Pericardial effusion, no. (%) | 379 | 65 (17.2) | 267 | 47 (17.6) | 112 | 18 (16.1) | 0.718 |
| TAPSE/sPAP, mm/mmHg | 166 | 0.36 (0.23-0.49) | 138 | 0.38 (0.24-0.49) | 28 | 0.29 (0.21-0.46) | 0.113 |
| Diastolic dysfunction, no. (%) | 300 | 132 (44.0) | 191 | 91 (47.6) | 109 | 41 (37.6) | 0.092 |
| mPAP, mmHg | 429 | 33 (26-43) | 289 | 32 (26-42) | 140 | 34 (26-45) | 0.106 |
| PAWP, mmHg | 429 | 9 (7-12) | 289 | 9 (7-12) | 140 | 9 (7-12) | 0.417 |
| PVR, WU | 429 | 5.2 (3.3-8.0) | 289 | 5.1 (3.2-7.6) | 140 | 5.5 (3.5-8.7) | 0.106 |
| CI, L/min/m^2^ | 398 | 2.7 (2.2-3.2) | 272 | 2.6 (2.2-3.2) | 126 | 2.7 (2.2-3.2) | 0.845 |
| Lower mPAP/PVR, no. (%) | 429 | 118 (27.4) | 289 | 82 (28.4) | 140 | 36 (25.7) | 0.563 |
| **Other characteristics** |  |  |  |  |  |  |  |
| Pre-existing treatment, no. (%) | 422 | 141 (33.4) | 288 | 83 (28.8) | 134 | 58 (43.3) | **0.003** |
| Upfront treatment, no. (%) | 422 | 245 (58.1) | 288 | 186 (64.6) | 134 | 59 (44.0) | **<0.001** |
| - Monotherapy, no. (%) | 422 | 159 (37.7) | 288 | 114 (39.6) | 134 | 45 (33.6) | 0.236 |
| - Combination, no. (%) | 422 | 86 (20.4) | 288 | 72 (25.0) | 134 | 14 (10.5) | **0.001** |
| Deaths, no. (%) | 429 | 172 (40.1) | 289 | 93 (32.2) | 140 | 79 (56.4) | **<0.001** |
| Lung transplants, no. (%) | 338 | 13 (3.9) | 227 | 11 (4.9) | 111 | 2 (1.8) | 0.172 |
| Dx after 2015, no. (%) | 429 | 237 (55.2) | 289 | 187 (64.7) | 140 | 50 (35.7) | **<0.001** |
| Observation time, years | 429 | 3.3 (1.4-5.6) | 289 | 3.1 (1.1-5.6) | 140 | 3.6 (1.9-5.7) | **0.041** |
| 1-, 3- and 5-year TFS (%) | 429 | 93/78/64 | 289 | 93/82/71 | 140 | 93/72/52 | **0.009** |

Data are presented as no. (%), mean ± SD or median (Q1-Q3) as appropriate. SSc: systemic sclerosis; lcSSc: limited cutaneous systemic sclerosis; PAH: pulmonary arterial hypertension; mRSS: modified Rodnan skin score; ACA: anti-centromere antibody; FVC: forced vital capacity; DLCO: diffusing capacity of the lung for carbon monoxide; 6MWD: 6-min walk distance; WHO-FC: World Health Organization functional class; ILD: interstitial lung disease, limited extent; NT-proBNP: N-terminal brain natriuretic peptide; TAPSE/sPAP: tricuspid annular plane systolic excursion/systolic pulmonary artery pressure; mPAP: mean pulmonary arterial pressure; PAWP: pulmonary arterial wedge pressure; PVR: pulmonary vascular resistance; RAP: right atrial pressure; CI: cardiac index; SVI: stroke volume index; SvO2: mixed-venous oxygen saturation; Dx: diagnosis; TFS: transplant-free survival. P-values represent pairwise comparisons.

**Supplementary Table S5:** Demographic and clinical characteristics of patients fulfilling the different risk stratification tools^a^.

|  | No. | ESC/ERS three- and four-strata  (n = 289) | No. | SPAHR updated (n = 413) | No. | REVEAL Lite 2  (n = 229) |
| --- | --- | --- | --- | --- | --- | --- |
| Age, years | 289 | 65 ± 11 | 413 | 65 ± 11 | 229 | 65 ± 11 |
| Male sex, no. (%) | 289 | 45 (15.6) | 413 | 59 (14.3) | 229 | 39 (17.0) |
| **SSc characteristics** |  |  |  |  |  |  |
| SSc duration, years | 268 | 8.7 (3.1-15.5) | 391 | 9.2 (3.6-16.4) | 212 | 7.7 (3.1-13.8) |
| lcSSc, no. (%) | 285 | 233 (81.8) | 406 | 333 (82.0) | 227 | 192 (84.6) |
| mRSS, median | 259 | 5.6 ± 6.6 | 353 | 4.5 ± 6.2 | 211 | 5.5 ± 6.3 |
| ACA, no. (%) | 289 | 177 (61.3) | 411 | 262 (63.8) | 229 | 143 (62.5) |
| Digital ulcers, no. (%) | 286 | 120 (42.0) | 409 | 166 (40.6) | 227 | 88 (38.8) |
| Teleangiectasia, no. (%) | 283 | 243 (85.9) | 402 | 340 (84.6) | 226 | 202 (89.4) |
| Joint synovitis, no. (%) | 259 | 43 (16.6) | 375 | 55 (14.7) | 199 | 32 (16.1) |
| Muscle weakness, no. (%) | 239 | 37 (15.5) | 347 | 56 (16.1) | 180 | 18 (10.0) |
| Renal crisis, no. (%) | 285 | 13 (4.6) | 395 | 16 (4.1) | 225 | 10 (4.4) |
| **Lung characteristics** |  |  |  |  |  |  |
| FVC, % predicted | 284 | 89.7 ± 21.2 | 393 | 91.2 ± 21.0 | 225 | 89.8 ± 20.3 |
| DLCO, % predicted | 271 | 43 (33-52) | 368 | 43 (33-52) | 220 | 43 (32-52) |
| 6MWD, m | 209 | 353 ± 131 | 306 | 341 ± 127 | 204 | 355 ± 131 |
| WHO-FC III and IV, no. (%) | 282 | 145 (51.4) | 405 | 205 (50.6) | 228 | 125 (54.8) |
| ILD, no. (%) | 289 | 146 (50.5) | 413 | 180 (43.6) | 229 | 113 (49.3) |
| **Heart characteristics** |  |  |  |  |  |  |
| NT-proBNP, ng/L | 256 | 568 (202-1495) | 257 | 567 (203-1485) | 205 | 629 (203-1599) |
| Right atrial area, cm^2^ | 99 | 18.0 (15.0-22.0) | 110 | 17.6 (15.0-22.0) | 87 | 17.5 (14.0-22.0) |
| Pericardial effusion, no. (%) | 267 | 47 (17.6) | 378 | 65 (17.2) | 223 | 38 (17.0) |
| TAPSE/sPAP, mm/mmHg | 138 | 0.38 (0.24-0.49) | 165 | 0.36 (0.23-0.49) | 105 | 0.34 (0.23-0.48) |
| Diastolic dysfunction, no. (%) | 191 | 91 (47.6) | 298 | 132 (44.3) | 140 | 72 (51.4) |
| mPAP, mmHg | 289 | 32 (26-42) | 413 | 33 (26-43) | 229 | 33 (26-43) |
| PAWP, mmHg | 289 | 9 (7-12) | 413 | 9 (7-12) | 229 | 9 (6-12) |
| PVR, WU | 289 | 5.3 (3.3- 8.1) | 413 | 5.3 (3.3-8.1) | 229 | 5.3 (3.3-7.9) |
| CI, L/min/m^2^ | 272 | 2.7 (2.2-3.2) | 385 | 2.7 (2.2-3.2) | 215 | 2.6 (2.2-3.2) |
| Lower mPAP/PVR, no. (%) | 289 | 82 (28.4) | 413 | 114 (27.6) | 229 | 59 (25.8) |
| **Other characteristics** |  |  |  |  |  |  |
| Pre-existing treatment, no. (%) | 288 | 83 (28.8) | 406 | 131 (32.3) | 228 | 52 (22.8) |
| Upfront treatment, no. (%) | 288 | 186 (64.6) | 406 | 240 (59.1) | 228 | 151 (66.2) |
| - Monotherapy, no. (%) | 288 | 114 (39.6) | 406 | 156 (38.4) | 228 | 93 (40.8) |
| - Combination, no. (%) | 288 | 72 (25.0) | 406 | 84 (20.7) | 228 | 58 (25.4) |
| Deaths, no. (%) | 289 | 93 (32.2) | 413 | 163 (39.7) | 229 | 81 (35.4) |
| Lung transplants, no. (%) | 227 | 11 (4.9) | 327 | 13 (4.0) | 173 | 11 (6.4) |
| Dx after 2015, no. (%) | 289 | 187 (64.7) | 413 | 231 (55.9) | 229 | 141 (61.6) |
| Observation time, years | 289 | 3.1 (1.1-5.7) | 413 | 3.3 (1.4-5.6) | 229 | 3.4 (1.5-5.9) |
| 1-, 3- and 5-year TFS (%) | 289 | 93/82/71 | 413 | 93/79/64 | 229 | 94/82/70 |

Data are presented as no. (%), mean ± SD or median (Q1-Q3) as appropriate. SSc: systemic sclerosis; lcSSc: limited cutaneous systemic sclerosis; PAH: pulmonary arterial hypertension; mRSS: modified Rodnan skin score; ACA: anti-centromere antibody; FVC: forced vital capacity; DLCO: diffusing capacity of the lung for carbon monoxide; 6MWD: 6-min walk distance; WHO-FC: World Health Organization functional class; ILD: interstitial lung disease, limited extent; NT-proBNP: N-terminal brain natriuretic peptide; TAPSE/sPAP: tricuspid annular plane systolic excursion/systolic pulmonary artery pressure; mPAP: mean pulmonary arterial pressure; PAWP: pulmonary arterial wedge pressure; PVR: pulmonary vascular resistance; RAP: right atrial pressure; CI: cardiac index; SVI: stroke volume index; SvO2: mixed-venous oxygen saturation; Dx: diagnosis; TFS: transplant-free survival. ^a^Statistical comparisons were not feasible due to overlapping populations across the four tools.

**Supplementary Table S6:** Proportion of patients in the different risk groups according to diagnostic period before and after 2015.

|  | All SSc-PAH | Before 2015 | After 2015 | *P* |
| --- | --- | --- | --- | --- |
| **ESC/ERS three-strata** |  |  |  |  |
| Low | 126 (44) | 46 (45) | 80 (43) | 0.704 |
| Intermediate | 153 (53) | 51 (50) | 102 (55) | 0.459 |
| High | 10 (3) | 5 (5) | 5 (3) | 0.322 |
| **ESC/ERS four-strata** |  |  |  |  |
| Low | 69 (24) | 24 (24) | 45 (24) | 0.919 |
| Intermediate-low | 94 (33) | 37 (36) | 57 (30) | 0.315 |
| Intermediate-high | 90 (31) | 25 (25) | 65 (35) | 0.072 |
| High | 36 (12) | 16 (17) | 20 (11) | 0.219 |
| **SPAHR updated** |  |  |  |  |
| Low | 170 (41) | 70 (38) | 100 (43) | 0.322 |
| Intermediate-low | 161 (39) | 72 (40) | 89 (39) | 0.831 |
| Intermediate-high | 67 (16) | 35 (19) | 32 (14) | 0.141 |
| High | 15 (4) | 5 (3) | 10 (4) | 0.394 |
| **REVEAL Lite 2** |  |  |  |  |
| Low | 101 (44) | 39 (44) | 62 (44) | 0.959 |
| Intermediate | 56 (24) | 22 (25) | 34 (24) | 0.879 |
| High | 72 (31) | 27 (31) | 45 (32) | 0.845 |

ESC/ERS: European Society of Cardiology and European Respiratory Society; SPAHR: Swedish Pulmonary Arterial Hypertension Registry; REVEAL: Registry to Evaluate Early and Long-Term PAH Disease Management. P-values represent pairwise comparisons.

**Supplementary Table S7:** Hazard ratios (HR) with 95% confidence intervals (CI) for all-cause mortality by risk groups in the four risk stratification tools in the total SSc-PAH cohort.

|  | HR (95% CI), p-value | | | |
| --- | --- | --- | --- | --- |
|  | ESC/ERS three-strata model  (n = 289) | ESC/ERS four-strata model  (n = 289) | SPAHR updated (n = 413) | REVEAL Lite 2  (n = 229) |
| Intermediate  (-low) vs. low | 2.79  (1.76, 4.41) **<0.001** | 1.42  (0.68, 2.94)  *0.352* | 2.13  (1.45, 3.11)  **<0.001** | 2.47  (1.29, 4.75)  **0.007** |
| Intermediate-high vs. low | N/A | 4.82  (2.43, 9.57)  **<0.001** | 4.72  (3.06, 7.27)  **<0.001** | N/A |
| High vs. low | 5.53  (2.26, 13.53)  **<0.001** | 9.01  (4.31, 18.81)  **<0.001** | 5.89  (2.74, 12.66)  **<0.001** | 7.84  (4.44, 13.86)  **<0.001** |
| Intermediate-high vs. intermediate-low | N/A | 3.41  (1.98, 5.87)  **<0.001** | 2.22  (1.51, 3.26)  **<0.001** | N/A |
| High vs. Intermediate (-low) | 1.98  (0.85, 4.61)  *0.112* | 6.36  (3.47, 11.67)  **<0.001** | 2.77  (1.32, 5.79)  **0.007** | 3.17  (1.81, 5.56)  **<0.001** |
| High vs. intermediate-high | N/A | 1.87  (1.10, 3.18)  **0.022** | 1.25  (0.58, 2.67)  *0.567* | N/A |

HR: hazard ratio; CI: confidence interval; ESC/ERS: European Society of Cardiology and European Respiratory Society; SPAHR: Swedish Pulmonary Arterial Hypertension Registry; REVEAL: Registry to Evaluate Early and Long-Term PAH Disease Management. P-values represent the significance of the hazard ratios obtained from univariable Cox regression analysis.

**Supplementary Figure S4:** Impact of WHO-FC, 6MWD, and NT-proBNP on predicting intermediate-high vs intermediate-low risk in univariable logistic regression (panel A), and comparison of these risk parameters using area under the ROC curve (AUC) (panel B).

**A**


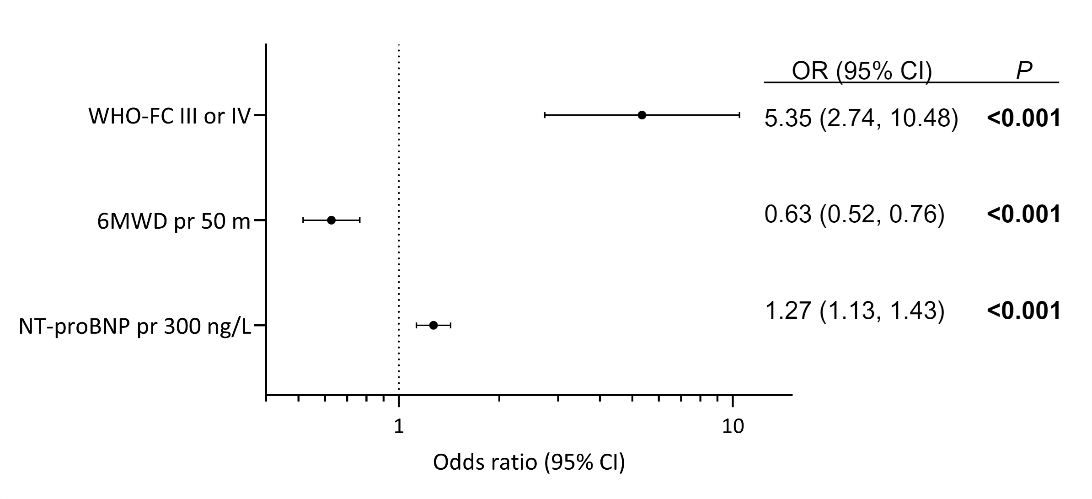


**B**


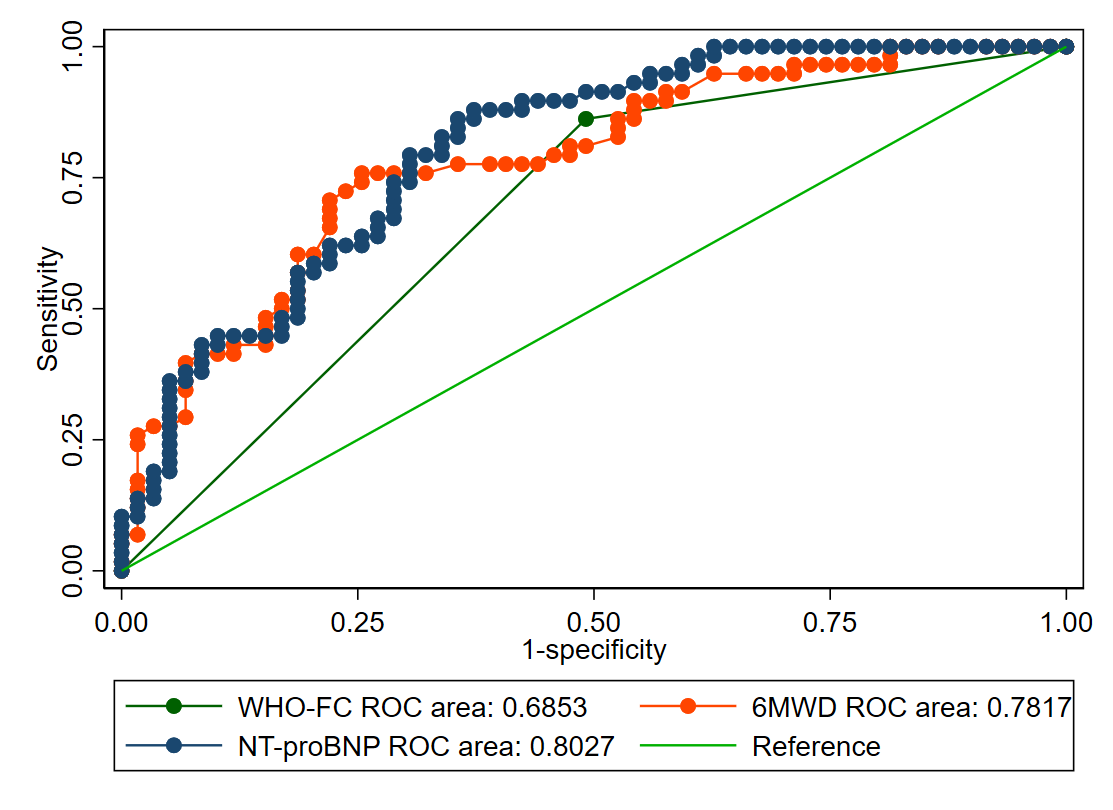


WHO-FC: World Health Organization functional class, 6MWD: 6-minute walk distance; NT-proBNP: N-terminal pro-brain natriuretic peptide. OR: odds ratio, CI: confidence interval.

**Panel A** shows the impact of WHO-FC, 6MWD, and NT-proBNP on predicting intermediate-high vs. intermediate-low risk using univariable logistic regression, with odds ratios and 95% confidence intervals (CI). ORs are presented for WHO-FC III and IV compared to WHO-FC I and II combined, for 6MWD per 50-meter increase, and for NT-proBNP per 300 ng/L increase.

**Panel B** compares the predictive abilities of these risk parameters using the area under the ROC curve (AUC) from univariable logistic regression. The p-values for differences in AUC between the parameters are: WHO-FC vs. 6MWD, p=0.55; WHO-FC vs. NT-proBNP, p=0.007; 6MWD vs. NT-proBNP, p=0.94. **Panel A** was created using BioRender.com.

**Supplementary Table S8:** Impact of general, SSc-specific, hemodynamic, and individual risk parameters on mortality in the total SSc-PAH cohort.

|  | No. | HR | 95% CI | *P* |
| --- | --- | --- | --- | --- |
| Age, years^a^ | 429 | 1.03 | 1.01, 1.04 | **<0.001** |
| Male sex^a^ | 429 | 1.15 | 0.75, 1.77 | 0.526 |
| SSc disease duration, years | 406 | 1.003 | 0.99, 1.02 | 0.673 |
| dcSSc | 420 | 1.21 | 0.82, 1.77 | 0.345 |
| mRSS | 361 | 1.01 | 0.98, 1.04 | 0.683 |
| ACA positive^a^ | 427 | 1.36 | 0.98, 1.89 | 0.067 |
| Digital ulcers | 423 | 1.07 | 0.79, 1.46 | 0.654 |
| Telangiectasia | 417 | 0.72 | 0.47, 1.10 | 0.130 |
| Joint synovitis | 387 | 1.06 | 0.67, 1.66 | 0.811 |
| Muscle weakness | 359 | 1.64 | 1.10, 2.45 | **0.016** |
| Renal crisis | 407 | 1.09 | 0.51, 2.32 | 0.825 |
| FVC% predicted | 408 | 0.99 | 0.98, 0.995 | **0.002** |
| DLCO% predicted^a^ | 382 | 0.97 | 0.96, 0.99 | **<0.001** |
| ILD^a^ | 429 | 1.08 | 0.79, 1.47 | 0.618 |
| Diastolic dysfunction | 300 | 1.68 | 1.15, 2.45 | **0.007** |
| Pre-existing treatment^a^ | 422 | 1.53 | 1.12, 2.09 | **0.007** |
|  |  |  |  |  |
| **Hemodynamics:** |  |  |  |  |
| mPAP≥25 and PVR>3 | 429 | 2.89 | 1.92, 4.37 | **<0.001** |
| mPAP, mmHg | 429 | 1.03 | 1.02, 1.04 | **<0.001** |
| PAWP, mmHg | 429 | 0.94 | 0.90, 0.99 | **0.009** |
| PVR, WU | 429 | 1.09 | 1.06, 1.12 | **<0.001** |
|  |  |  |  |  |
| **Risk parameters:** |  |  |  |  |
| Signs of right heart failure | 106 | 3.94 | 2.26, 6.85 | **<0.001** |
| Progression of symptoms | 105 |  |  |  |
| - No (ref) |  | 1 |  |  |
| - Slow |  | 1.22 | 0.58, 2.59 | 0.596 |
| - Rapid |  | 6.36 | 2.70, 14.99 | **<0.001** |
| Syncopy^b^ | 105 | - | - | - |
| WHO-FC III or IV vs. I or II | 418 | 2.45 | 1.78, 3.36 | **<0.001** |
| 6MWD, m | 306 | 0.99 | 0.991, 0.995 | **<0.001** |
| Peak VO2^b^ | 5 | - | - | - |
| NT-proBNP, ng/L | 260 | 1.0001 | 1.00006, 1.0001 | **<0.001** |
| BNP, ng/L | 38 | 1.0005 | 0.999, 1.001 | 0.167 |
| RA area, cm^2^ | 111 | 1.08 | 1.03, 1.14 | **0.004** |
| TAPSE/sPAP, mm/mmHg | 166 | 0.02 | 0.002, 0.143 | **<0.001** |
| Pericardial effusion | 379 | 1.54 | 1.06, 2.25 | **0.025** |
| cMRI RVEF, % | 21 | 0.97 | 0.93, 1.02 | 0.245 |
| cMRI RVESVI, mL/m^2^ | 19 | 1.01 | 0.995, 0.102 | 0.243 |
| cMRI SVI, mL/m^2^ | 22 | 0.98 | 0.91, 1.06 | 0.684 |
| RAP, mmHg | 100 | 1.05 | 0.99, 1.12 | 0.126 |
| CI, L/min/m^2^ | 398 | 0.71 | 0.57, 0.87 | **0.001** |
| SVI at RHC, mL/m^2^ | 102 | 0.97 | 0.94, 0.99 | **0.013** |
| SvO2, % | 100 | 0.94 | 0.92, 0.96 | **<0.001** |
| Systolic blood pressure, mmHg | 105 | 0.99 | 0.97, 1.00 | 0.080 |
| Heart rate, beats/min | 103 | 0.997 | 0.98, 1.02 | 0.776 |
| eGFR, mL/min/1.73m^2^ | 105 | 0.99 | 0.979, 0.998 | **0.022** |

HR: hazard ratio; CI: confidence interval; SSc: systemic sclerosis; dcSSc: diffuse cutaneous systemic sclerosis; mRSS: modified Rodnan skin score; ACA: anti-centromere antibody; FVC: forced vital capacity; DLCO: diffusing capacity of the lung for carbon monoxide; ILD: interstitial lung disease; mPAP: mean pulmonary arterial pressure; PAWP: pulmonary arterial wedge pressure; PVR: pulmonary vascular resistance; WHO-FC: World Health Organization functional class; 6MWD: 6-min walk distance; VO2: oxygen uptake; NT-proBNP: N-terminal brain natriuretic peptide; RA area: right atrial area; TAPSE/sPAP: tricuspid annular plane systolic excursion/systolic pulmonary artery pressure; cMRI: Cardiac magnetic resonance imaging; RVEF, right ventricular ejection fraction; RVESVI, right ventricular end-systolic volume index; SVI, stroke volume index; RAP: right atrial pressure; CI: cardiac index; SvO2: mixed-venous oxygen saturation, eGFR: estimated Glomerular Filtration Rate. ^a^Included in the multivariable Cox regression models; ^b^Too few occurences to calculate HR. P-values represent the significance of the hazard ratios obtained from univariable Cox regression analysis.

**Supplementary Table S9:** Performance of the adjusted risk stratification tools in predicting all-cause mortality compared to the corresponding univariable tool in the total SSc-PAH cohort.

| Risk stratification tool | AUC Univariable (95% CI) | AUC Multivariable (95% CI) | *P* |
| --- | --- | --- | --- |
| ESC/ERS three-strata | 0.63 (0.57, 0.70) | 0.67 (0.61, 0.74) | 0.087 |
| ESC/ERS four-strata | 0.71 (0.64, 0.77) | 0.72 (0.65, 0.78) | 0.524 |
| SPAHR updated | 0.65 (0.59, 0.70) | 0.65 (0.59, 0.71) | 0.906 |
| REVEAL Lite 2 | 0.70 (0.63, 0.77) | 0.72 (0.65, 0.79) | 0.586 |

ESC/ERS: European Society of Cardiology and European Respiratory Society; SPAHR: Swedish Pulmonary Arterial Hypertension Registry; REVEAL: Registry to Evaluate Early and Long-Term PAH Disease Management; AUC: area under the ROC curve; CI: confidence interval.

The risk stratification tools were adjusted for age, male sex, pre-existing vascular-targeted therapy, DLCO% predicted, ILD of limited extent, and anti-centromere antibodies. Predictive abilities were evaluated using area under the ROC curve (AUC), and performance was compared to the corresponding univariable tool. P-values represent the statistical significance of differences in predictive performance between the univariable and multivariable models.

**Supplementary Figure S5: A**, Proportion of patients; and **B**, observed 1-year mortality across risk categories in the four risk stratification tools for treatment-naïve patients.

**A**

**
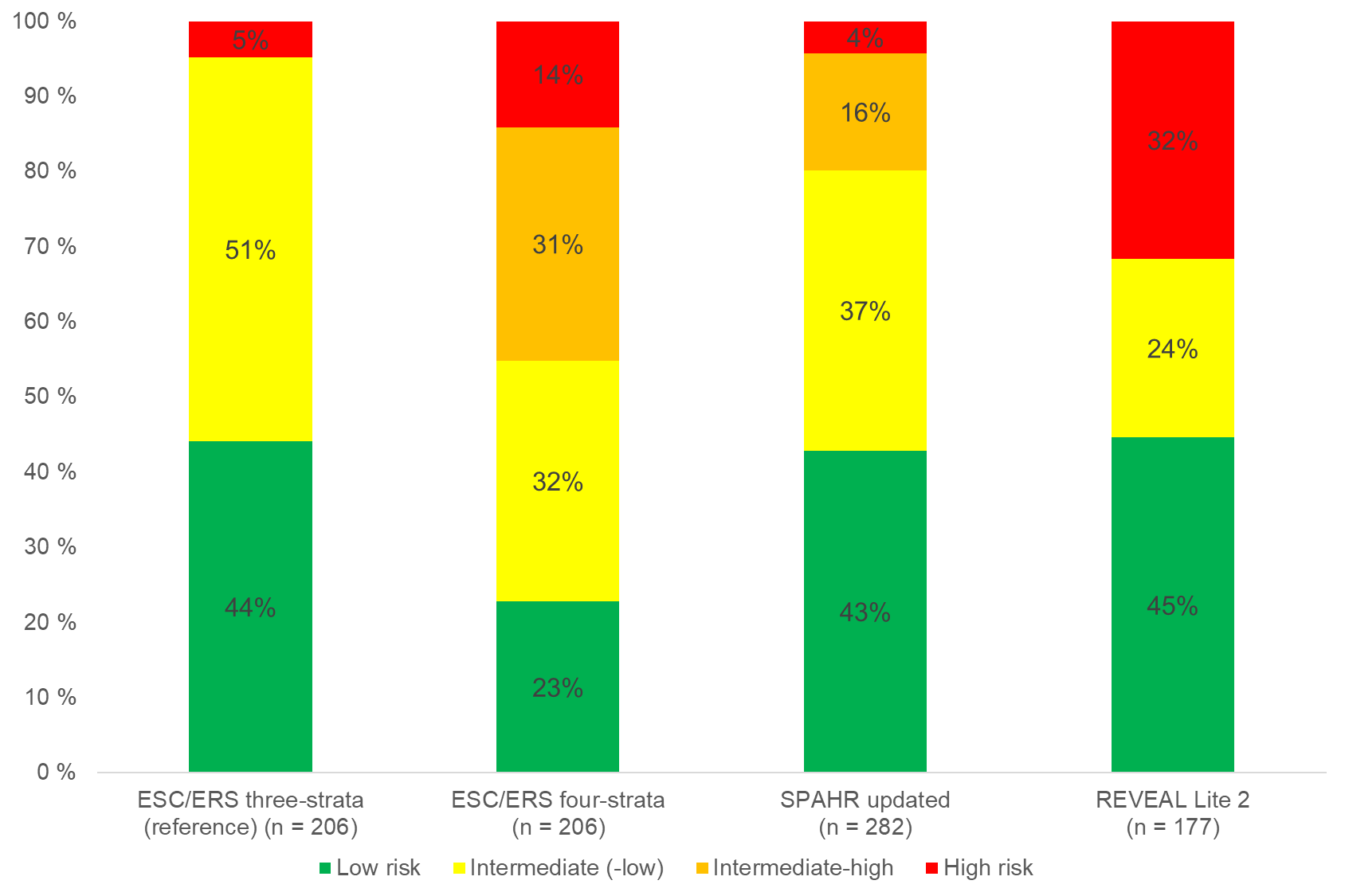
**

**B**

**
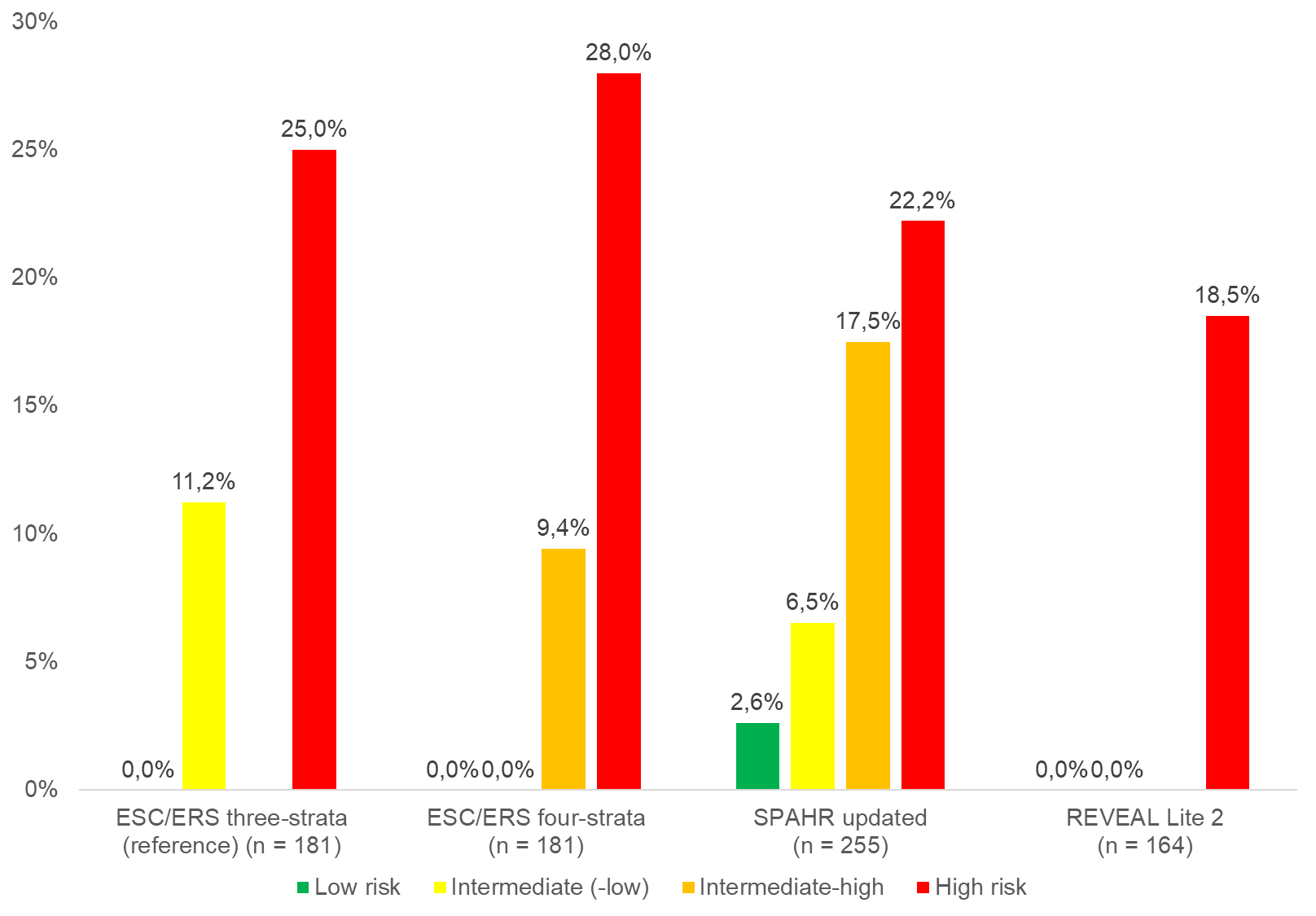
**

ESC/ERS: European Society of Cardiology and European Respiratory Society; SPAHR: Swedish Pulmonary Arterial Hypertension Registry; REVEAL: Registry to Evaluate Early and Long-Term PAH Disease Management. The 1-year mortality rate was determined for patients who were either deceased or had at least a one-year observation period.

**Supplementary Figure S6:** Transplant-free survival by risk groups in the four risk stratification tools for treatment-naïve patients.

***
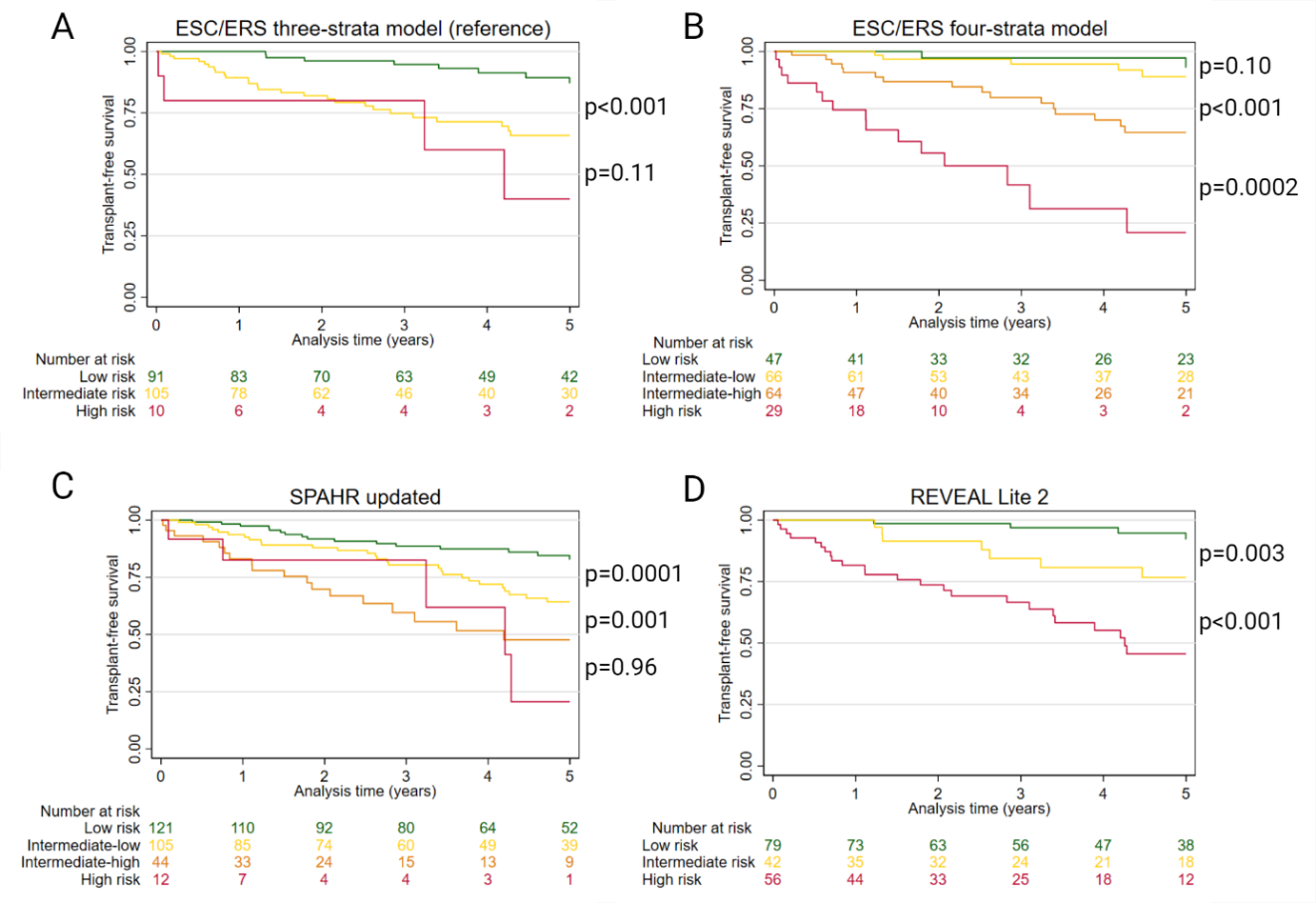
***

ESC/ERS: European Society of Cardiology and European Respiratory Society; SPAHR: Swedish Pulmonary Arterial Hypertension Registry; REVEAL: Registry to Evaluate Early and Long-Term PAH Disease Management.

**A**, ESC/ERS three-strata model; **B**, ESC/ERS four-strata model; **C**, “SPAHR updated”; and **D**, “REVEAL Lite 2”. P-values for pairwise comparison of the risk groups using the log-rank test.

**Supplementary Table S10:** Hazard ratios (HR) with 95% confidence intervals (CI) for all-cause mortality by risk groups in the four risk stratification tools for treatment-naïve patients.

|  | HR (95% CI), p-value | | | |
| --- | --- | --- | --- | --- |
|  | ESC/ERS three-strata model (reference)  (n = 206) | ESC/ERS four-strata model  (n = 206) | SPAHR updated (n = 282) | REVEAL Lite 2  (n = 177) |
| Intermediate  (-low) vs. low | 3.71  (2.09, 6.59) **<0.001** | 2.34  (0.83, 6.58)  *0.108* | 2.47  (1.54, 3.97)  **0.001** | 3.01  (1.39, 6.52)  **0.005** |
| Intermediate-high vs. low | N/A | 10.19  (3.74, 27.79)  **<0.001** | 5.79  (3.29, 10.20)  **<0.001** | N/A |
| High vs. low | 7.84  (3.03, 20.33)  **<0.001** | 35.31  (11.88, 104.91)  **<0.001** | 6.48  (2.63, 15.96)  **<0.001** | 12.75  (6.10, 26.69)  **<0.001** |
| Intermediate-high vs. intermediate-low | N/A | 4.35  (2.22, 8.55)  **<0.001** | 2.34  (1.42, 3.86)  **0.001** | N/A |
| High vs. intermediate  (-low) | 2.11  (0.89, 5.02)  *0.089* | 15.09  (6.83, 33.37)  **<0.001** | 2.62  (1.11, 6.21)  **0.028** | 4.24  (2.16, 8.32)  **<0.001** |
| High vs. intermediate-high | N/A | 3.46  (1.84, 6.54)  **<0.001** | 1.12  (0.46, 2.74)  *0.803* | N/A |

HR: hazard ratio; CI: confidence interval; AUC: area under the ROC curve; ESC/ERS: European Society of Cardiology and European Respiratory Society; SPAHR: Swedish Pulmonary Arterial Hypertension Registry; REVEAL: Registry to Evaluate Early and Long-Term PAH Disease Management; N/A: not applicable. P-values represent the significance of the hazard ratios obtained from univariable Cox regression analysis.

**Supplementary Table S11:** Performance of the risk stratification tools in predicting all-cause mortality compared to the ESC/ERS three-strata model (reference) in unadjusted analysis for treatment-naïve patients.

| Comparison | Tool | AUC (95% CI) | *P* |
| --- | --- | --- | --- |
| ESC/ERS three-strata vs. ESC/ERS four-strata | ESC/ERS three-strata (reference) | 0.64 (0.57, 0.71) | **0.013** |
|  | ESC/ERS four-strata | 0.72 (0.65, 0.79) |  |
| ESC/ERS three-strata vs. SPAHR updated | ESC/ERS three-strata (reference) | 0.64 (0.57, 0.71) | 0.066 |
|  | SPAHR updated | 0.68 (0.61, 0.75) |  |
| ESC/ERS three-strata vs. REVEAL Lite 2 | ESC/ERS three-strata (reference) | 0.66 (0.58, 0.73) | **0.028** |
|  | REVEAL Lite 2 | 0.73 (0.66, 0.81) |  |

AUC: area under the ROC curve; CI: confidence interval; ESC/ERS: European Society of Cardiology and European Respiratory Society; SPAHR: Swedish Pulmonary Arterial Hypertension Registry; REVEAL: Registry to Evaluate Early and Long-Term PAH Disease Management.

Predictive abilities were evaluated using area under the ROC curve (AUC) based on univariable Cox regression analysis, and performance was compared to the ESC/ERS three-strata model (reference). P-values represent the statistical significance of differences in predictive performance between the risk stratification tools.

**Supplementary Figure S7:** Impact of risk stratification tools on predicting all-cause mortality in multivariable analysis for treatment-naïve patients.

**
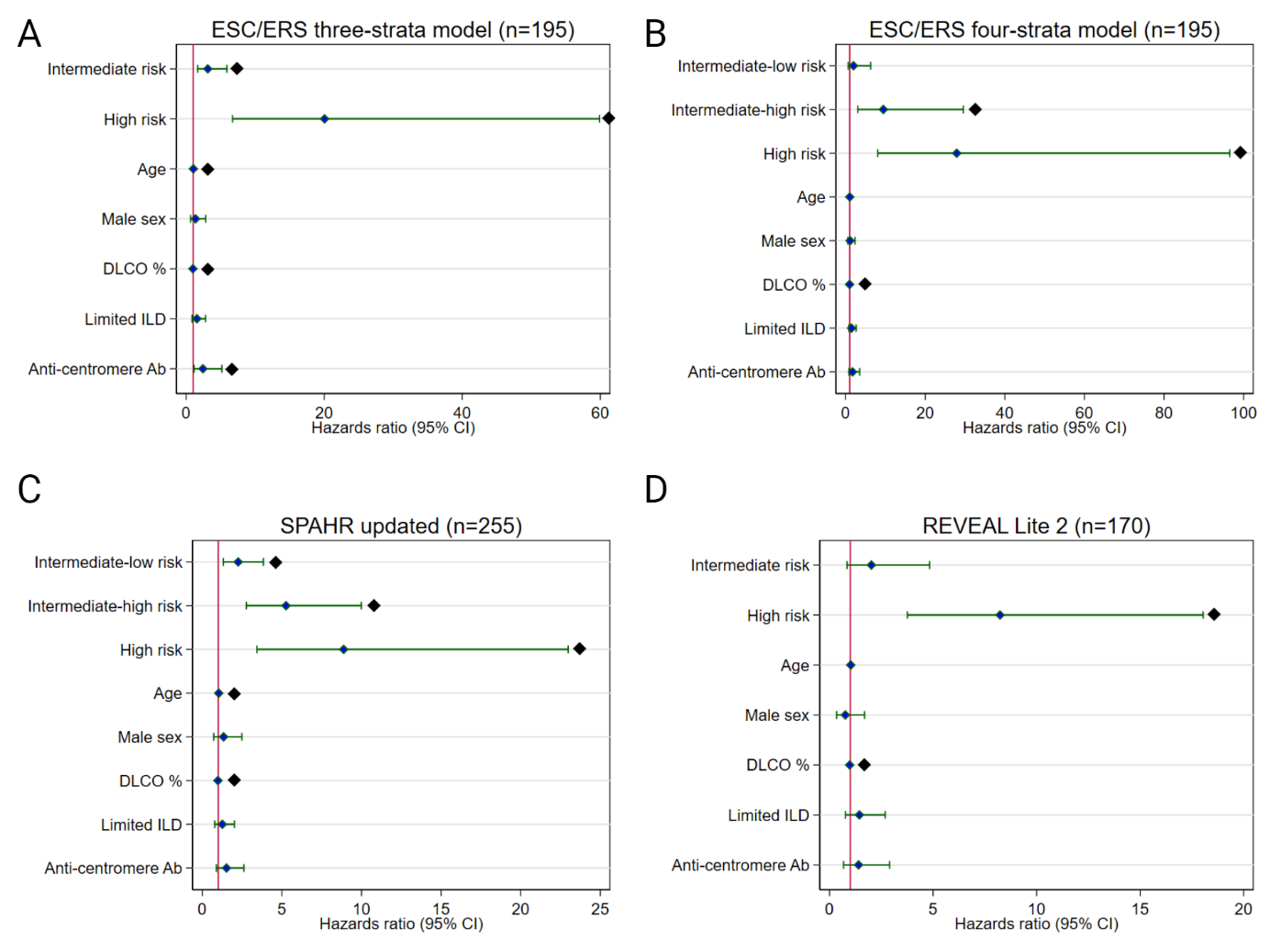
**

ESC/ERS: European Society of Cardiology and European Respiratory Society; SPAHR: Swedish Pulmonary Arterial Hypertension Registry; REVEAL: Registry to Evaluate Early and Long-Term PAH Disease Management; DLCO: diffusing capacity of the lung for carbon monoxide; ILD: interstitial lung disease, limited extent; Ab: antibodies; CI: confidence interval.

**A**, ESC/ERS three-strata model (reference); **B**, ESC/ERS four-strata model; **C**, “SPAHR updated”; and **D**, “REVEAL Lite 2”.

The multivariable models are adjusted for age, male sex, DLCO% predicted, ILD of limited extent, and anti-centromere antibodies, with hazard ratios (HR) and 95% confidence intervals (CI) shown for all variables. Hazard ratios of risk groups are referenced to the low-risk group. ♦= indicates p <0.05.

**Supplementary Table S12:** Performance of the adjusted risk stratification tools in predicting all-cause mortality compared to the corresponding univariable tool for treatment-naïve patients.

| Risk stratification tool | AUC Univariable (95% CI) | AUC Multivariable (95% CI) | *P* |
| --- | --- | --- | --- |
| ESC/ERS three-strata | 0.66 (0.59, 0.73) | 0.72 (0.64, 0.79) | 0.051 |
| ESC/ERS four-strata | 0.73 (0.66, 0.80) | 0.75 (0.68, 0.82) | 0.187 |
| SPAHR updated | 0.66 (0.60, 0.73) | 0.68 (0.61, 0.74) | 0.469 |
| REVEAL Lite 2 | 0.72 (0.64, 0.79) | 0.74 (0.66, 0.82) | 0.281 |

ESC/ERS: European Society of Cardiology and European Respiratory Society; SPAHR: Swedish Pulmonary Arterial Hypertension Registry; REVEAL: Registry to Evaluate Early and Long-Term PAH Disease Management; AUC: area under the ROC curve; CI: confidence interval.

The risk stratification tools were adjusted for age, male sex, pre-existing vascular-targeted therapy, DLCO% predicted, ILD of limited extent, and anti-centromere antibodies. Predictive abilities were evaluated using area under the ROC curve (AUC), and performance was compared to the corresponding univariable tool. P-values represent the statistical significance of differences in predictive performance between the univariable and multivariable models.

**Supplementary Table S13:** Proportion of patients in the different risk groups according to mPAP and PVR threshold groups.

|  | All SSc-PAH | mPAP 21-24/PVR 2-3 | mPAP ≥25 and PVR>3 | *P* |
| --- | --- | --- | --- | --- |
| **ESC/ERS three-strata** |  |  |  |  |
| Low | 126 (44) | 61 (74) | 65 (31) | <0.001 |
| Intermediate | 153 (53) | 20 (24) | 133 (64) | <0.001 |
| High | 10 (3) | 1 (1) | 9 (4) | 0.190 |
| **ESC/ERS four-strata** |  |  |  |  |
| Low | 69 (24) | 36 (44) | 33 (16) | <0.001 |
| Intermediate-low | 94 (33) | 30 (37) | 64 (31) | 0.354 |
| Intermediate-high | 90 (31) | 13 (16) | 77 (37) | <0.001 |
| High | 36 (12) | 3 (4) | 33 (16) | 0.004 |
| **SPAHR updated** |  |  |  |  |
| Low | 170 (41) | 82 (72) | 88 (29) | <0.001 |
| Intermediate-low | 161 (39) | 25 (22) | 136 (45) | <0.001 |
| Intermediate-high | 67 (16) | 7 (6) | 60 (20) | 0.001 |
| High | 15 (4) | 0 | 15 (5) | 0.015 |
| **REVEAL Lite 2** |  |  |  |  |
| Low | 101 (44) | 40 (68) | 61 (36) | <0.001 |
| Intermediate | 56 (24) | 13 (22) | 43 (25) | 0.616 |
| High | 72 (31) | 6 (10) | 66 (39) | <0.001 |

ESC/ERS: European Society of Cardiology and European Respiratory Society; SPAHR: Swedish Pulmonary Arterial Hypertension Registry; REVEAL: Registry to Evaluate Early and Long-Term PAH Disease Management; mPAP: mean pulmonary arterial pressure; PVR: pulmonary vascular resistance; SSc-PAH: systemic sclerosis-associated pulmonary arterial hypertension. P-values represent pairwise comparisons.

**Supplementary Figure S8:** Transplant-free survival in the higher vs. lower mPAP and PVR threshold groups in the total SSc-PAH cohort.
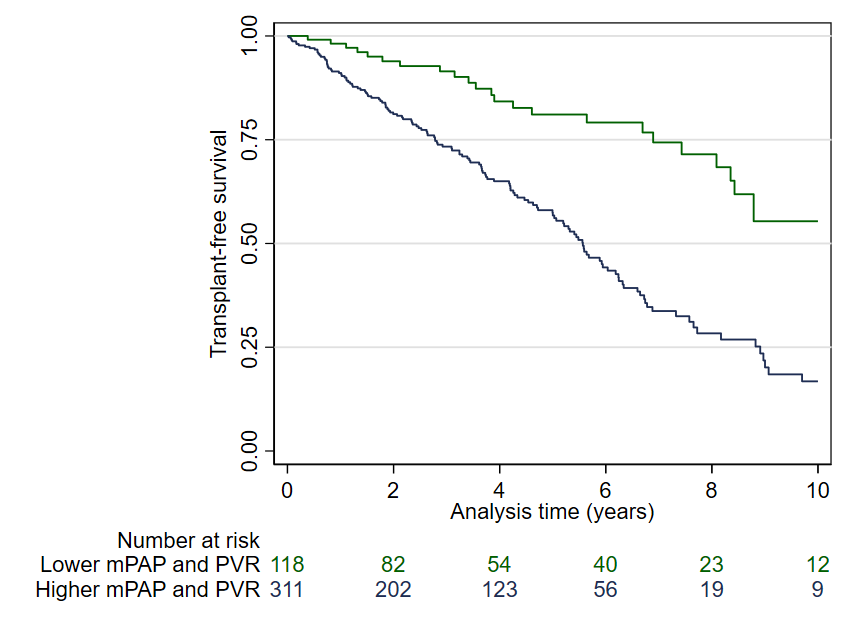


mPAP: mean pulmonary arterial pressure; PVR: pulmonary vascular resistance. Log-rank p-values represents the statistical comparison of survival curves between the groups (log-rank p<0.001).

**Supplementary Table S14:** Characteristics of patients according to mPAP and PVR threshold groups.

|  | No. | All SSc-PAH  (n = 429) | mPAP 21-24/PVR 2-3 (n = 118) | mPAP ≥25 and PVR>3 (n = 311) | *P* |
| --- | --- | --- | --- | --- | --- |
| Age, years | 429 | 65 ± 11 | 66 ± 11 | 65 ± 11 | 0.381 |
| Male sex, no. (%) | 429 | 60 (14.0) | 14 (11.9) | 46 (14.8) | 0.435 |
| **SSc characteristics** |  |  |  |  |  |
| SSc duration, years | 406 | 9.8 (3.7-16.5) | 12.6 (4.6-20.5) | 8.6 (3.2-15.4) | **0.003** |
| lcSSc, no. (%) | 420 | 342 (81.4) | 88 (75.2) | 254 (83.8) | **0.042** |
| mRSS, mean | 361 | 4.4 ± 6.2 | 5.6 ± 6.9 | 3.9 ± 5.8 | **0.019** |
| ACA positive, no. (%) | 427 | 273 (64.0) | 69 (58.5) | 204 (66.0) | 0.147 |
| Digital ulcers, no. (%) | 423 | 170 (40.2) | 50 (44.3) | 120 (38.7) | 0.304 |
| Teleangiectasia, no. (%) | 417 | 352 (84.4) | 91 (80.5) | 261 (85.9) | 0.183 |
| Joint synovitis, no. (%) | 387 | 57 (14.7) | 22 (20.8) | 35 (12.5) | **0.040** |
| Muscle weakness, no. (%) | 359 | 60 (16.7) | 22 (23.7) | 38 (14.3) | **0.037** |
| Renal crisis, no. (%) | 407 | 16 (3.9) | 7 (6.3) | 9 (3.1) | 0.138 |
| **Lung characteristics** |  |  |  |  |  |
| FVC, % predicted | 408 | 91.3 ± 21.1 | 95.6 ± 20.2 | 89.6 ± 21.2 | **0.009** |
| DLCO, % predicted | 382 | 43 (33-52) | 52 (43-61) | 40 (31-49) | **<0.001** |
| 6MWD, m | 306 | 341 ± 127 | 397 ± 117 | 322 ± 125 | **<0.001** |
| WHO-FC III and IV, no. (%) | 418 | 211 (50.5) | 30 (26.6) | 181 (59.3) | **<0.001** |
| ILD, no. (%) | 429 | 187 (43.6) | 46 (39.0) | 141 (45.3) | 0.236 |
| **Heart characteristics** |  |  |  |  |  |
| NT-proBNP, ng/L | 260 | 568 (203-1495) | 275 (136-608) | 866 (280-2056) | **<0.001** |
| Right atrial area, cm^2^ | 111 | 17.6 (14.9-22.0) | 16.6 (13.4-19.0) | 18.5 (15.0-23.8) | **0.012** |
| Pericardial effusion, no. (%) | 379 | 65 (17.2) | 8 (7.8) | 57 (20.6) | **0.004** |
| TAPSE/sPAP, mm/mmHg | 166 | 0.36 (0.23-0.49) | 0.55 (0.47-0.74) | 0.29 (0.22-0.42) | **<0.001** |
| Diastolic dysfunction, no. (%) | 300 | 132 (44.0) | 32 (39.0) | 100 (45.9) | 0.287 |
| mPAP, mmHg | 429 | 33 (26-43) | 23 (22-25) | 38 (30-46) | **<0.001** |
| PAWP, mmHg | 429 | 9 (7-12) | 10 (8-13) | 9 (6-12) | **0.001** |
| PVR, WU | 429 | 5.2 (3.3-8.0) | 2.7 (2.4-3.1) | 6.6 (4.6-9.4) | **<0.001** |
| CI, L/min/m^2^ | 398 | 2.7 (2.2-3.2) | 2.9 (2.4-3.5) | 2.6 (2.1-3.0) | **0.0001** |
| **Other characteristics** |  |  |  |  |  |
| Pre-existing treatment, no. (%) | 422 | 141 (33.4) | 33 (29.5) | 108 (34.8) | 0.301 |
| Upfront treatment, no. (%) | 422 | 245 (58.1) | 34 (30.4) | 211 (68.1) | **<0.001** |
| - Monotherapy, no. (%) | 422 | 159 (37.7) | 29 (25.9) | 130 (41.9) | **0.003** |
| - Combination, no. (%) | 422 | 86 (20.4) | 5 (4.5) | 81 (26.1) | **<0.001** |
| Deaths, no. (%) | 429 | 172 (40.1) | 28 (23.7) | 144 (46.3) | **<0.001** |
| Lung transplants, no. (%) | 338 | 13 (3.9) | 0 | 13 (5.0) | **0.048** |
| Dx after 2015, no. (%) | 429 | 237 (55.2) | 69 (58.5) | 168 (54.0) | 0.407 |
| Observation time, years | 429 | 3.3 (1.4-5.6) | 3.7 (1.5-7.0) | 3.1 (1.3-5.4) | **0.022** |

Data are presented as no. (%), mean ± SD or median (Q1-Q3) as appropriate. SSc: systemic sclerosis; lcSSc: limited cutaneous systemic sclerosis; PAH: pulmonary arterial hypertension; mRSS: modified Rodnan skin score; ACA: anti-centromere antibody; FVC: forced vital capacity; DLCO: diffusing capacity of the lung for carbon monoxide; 6MWD: 6-min walk distance; WHO-FC: World Health Organization functional class; ILD: interstitial lung disease, limited extent; NT-proBNP: N-terminal brain natriuretic peptide; TAPSE/sPAP: tricuspid annular plane systolic excursion/systolic pulmonary artery pressure; mPAP: mean pulmonary arterial pressure; PAWP: pulmonary arterial wedge pressure; PVR: pulmonary vascular resistance; RAP: right atrial pressure; CI: cardiac index; SVI: stroke volume index; SvO2: mixed-venous oxygen saturation; Dx: diagnosis; TFS: transplant-free survival. P-values represent pairwise comparisons.

**Supplementary Table S15:** Characteristics of patients in the lower mPAP and PVR threshold group segregated by low- or intermediate-low vs. intermediate-high or high-risk assessed by the ESC/ERS four-strata model.

|  | No. | mPAP 21-24/PVR 2-3  (n = 82) | Low- or intermediate-low (n = 66) | Intermediate-high or high  (n = 16) | *P* |
| --- | --- | --- | --- | --- | --- |
| Age, years | 82 | 67 ± 10 | 66 ± 10 | 71 ± 11 | 0.075 |
| Male sex, no. (%) | 82 | 12 (14.6) | 10 (15.2) | 2 (12.5) | 0.788 |
| **SSc characteristics** |  |  |  |  |  |
| SSc duration, years | 72 | 11.3 (4.4-20.9) | 10.8 (3.8-19.6) | 12.5 (8.8-25.0) | 0.159 |
| lcSSc, no. (%) | 82 | 60 (73.2) | 49 (74.2) | 11 (68.8) | 0.656 |
| mRSS, mean | 76 | 7.1 ± 7.2 | 6.2 ± 6.5 | 10.6 ± 9.1 | **0.034** |
| ACA positive, no. (%) | 82 | 47 (57.3) | 37 (56.1) | 10 (62.5) | 0.640 |
| Digital ulcers, no. (%) | 80 | 34 (42.5) | 25 (39.1) | 9 (56.3) | 0.214 |
| Teleangiectasia, no. (%) | 79 | 64 (81.0) | 50 (79.4) | 14 (87.5) | 0.459 |
| Joint synovitis, no. (%) | 74 | 16 (21.6) | 12 (20.3) | 4 (26.7) | 0.595 |
| Muscle weakness, no. (%) | 62 | 13 (21.0) | 9 (17.7) | 4 (36.4) | 0.167 |
| Renal crisis, no. (%) | 81 | 7 (8.6) | 3 (4.6) | 4 (25.0) | **0.009** |
| **Lung characteristics** |  |  |  |  |  |
| FVC, % predicted | 81 | 94.5 ± 19.6 | 94.0 ± 19.8 | 96.8 ± 19.2 | 0.604 |
| DLCO, % predicted | 77 | 52 (42-59) | 52 (45-61) | 41 (31-52) | **0.024** |
| 6MWD, m | 55 | 410 ± 120 | 439 ± 99 | 260 ± 108 | **<0.001** |
| WHO-FC III and IV, no. (%) | 79 | 22 (27.9) | 12 (18.8) | 10 (66.7) | **<0.001** |
| ILD, no. (%) | 82 | 37 (45.1) | 30 (45.5) | 7 (43.8) | 0.902 |
| **Heart characteristics** |  |  |  |  |  |
| NT-proBNP, ng/L | 78 | 270 (135-569) | 202 (123-389) | 1703 (691-3949) | **<0.001** |
| Right atrial area, cm^2^ | 25 | 17.0 (13.6-19.7) | 16.9 (13.3-19.9) | 17.6 | 0.720 |
| Pericardial effusion, no. (%) | 74 | 7 (9.5) | 7 (9.7) | 1 (3.6) | 0.566 |
| TAPSE/sPAP, mm/mmHg | 33 | 0.55 (0.47-0.74) | 0.58 (0.48-0.74) | 0.52 (0.39-0.70) | 0.352 |
| Diastolic dysfunction, no. (%) | 51 | 23 (45.1) | 18 (42.9) | 5 (55.6) | 0.487 |
| mPAP, mmHg | 82 | 23 (22-25) | 24 (22-25) | 23 (22-25) | 0.887 |
| PAWP, mmHg | 82 | 10 (8-12) | 10 (8-12) | 11 (8-13) | 0.635 |
| PVR, WU | 82 | 2.7 (2.3-3.1) | 2.7 (2.3-3.1) | 2.7 (2.4-3.2) | 0.495 |
| CI, L/min/m^2^ | 79 | 2.9 (2.4-3.5) | 3.0 (2.4-3.5) | 2.5 (2.2-3.5) | 0.265 |
| **Other characteristics** |  |  |  |  |  |
| Pre-existing treatment, no. (%) | 81 | 22 (27.2) | 16 (24.4) | 6 (40.0) | 0.216 |
| Upfront treatment, no. (%) | 81 | 27 (33.3) | 22 (33.3) | 5 (33.3) | 1.000 |
| - Monotherapy, no. (%) | 81 | 22 (27.2) | 19 (28.8) | 3 (20.0) | 0.490 |
| - Combination, no. (%) | 81 | 5 (6.2) | 3 (4.6) | 2 (13.3) | 0.202 |
| Deaths, no. (%) | 82 | 16 (19.5) | 10 (15.2) | 6 (37.5) | **0.043** |
| Lung transplants, no. (%) | 53 | 0 | 0 | 0 | N/A |
| Dx after 2015, no. (%) | 82 | 56 (68.3) | 44 (66.7) | 12 (75.0) | 0.520 |
| Observation time, years | 82 | 3.6 (1.3-6.5) | 3.7 (1.5-6.9) | 2.5 (0.4-5.4) | 0.098 |
| 1-, 3- and 5-year TFS (%) | 82 | 99/90/84 | 100/90/87 | 91/91/68 | **0.002** |

Data are presented as no. (%), mean ± SD or median (Q1-Q3) as appropriate. SSc: systemic sclerosis; lcSSc: limited cutaneous systemic sclerosis; PAH: pulmonary arterial hypertension; mRSS: modified Rodnan skin score; ACA: anti-centromere antibody; FVC: forced vital capacity; DLCO: diffusing capacity of the lung for carbon monoxide; 6MWD: 6-min walk distance; WHO-FC: World Health Organization functional class; ILD: interstitial lung disease, limited extent; NT-proBNP: N-terminal brain natriuretic peptide; TAPSE/sPAP: tricuspid annular plane systolic excursion/systolic pulmonary artery pressure; mPAP: mean pulmonary arterial pressure; PAWP: pulmonary arterial wedge pressure; PVR: pulmonary vascular resistance; RAP: right atrial pressure; CI: cardiac index; SVI: stroke volume index; SvO2: mixed-venous oxygen saturation; Dx: diagnosis; TFS: transplant-free survival. P-values represent pairwise comparisons.

**Supplementary Figure S9:** Impact of risk stratification tools on predicting all-cause mortality in multivariable analysis, including mPAP and PVR threshold groups, in the total SSc-PAH cohort.


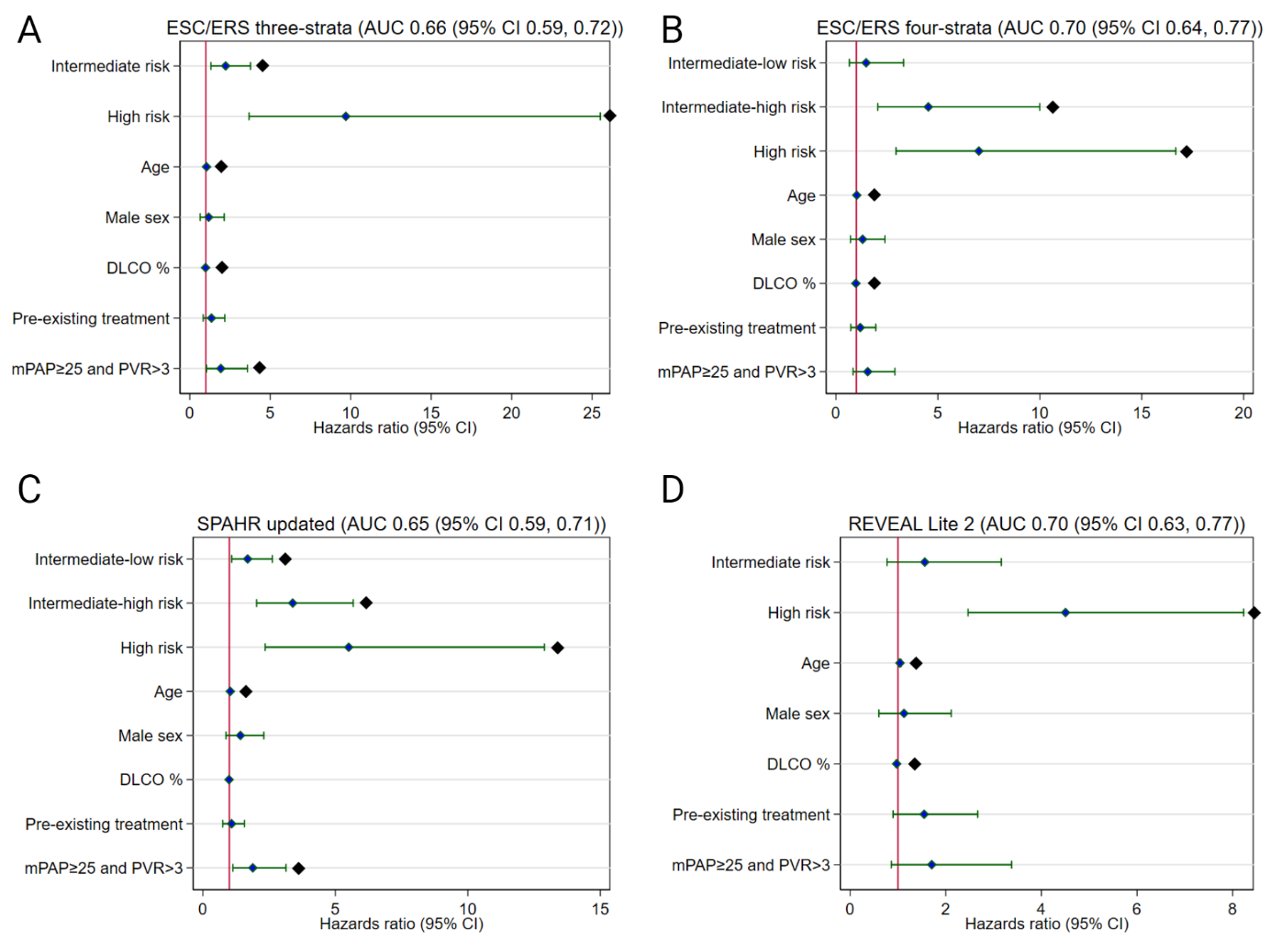


ESC/ERS: European Society of Cardiology and European Respiratory Society; SPAHR: Swedish Pulmonary Arterial Hypertension Registry; REVEAL: Registry to Evaluate Early and Long-Term PAH Disease Management; DLCO: diffusing capacity of the lung for carbon monoxide; mPAP: mean pulmonary arterial pressure; PVR: pulmonary vascular resistance; HR: hazard ratio; CI: confidence interval; AUC: area under the ROC curve.

**A**, ESC/ERS three-strata model (reference); **B**, ESC/ERS four-strata model; **C**, “SPAHR updated”; and **D**, “REVEAL Lite 2”.

The multivariable models are adjusted for age, male sex, DLCO% predicted, pre-existing vascular-targeted therapy, and mPAP and PVR threshold groups, with hazard ratios (HR) and 95% confidence intervals (CI) shown for all variables. Hazard ratios of risk groups are referenced to the low-risk group. ♦= indicates p <0.05.

**Supplementary Figure S10:** **A**, Proportion of patients; and **B**, observed 1-year mortality across risk categories in the four risk stratification tools in patients fulfilling all the four risk stratification tools.

**A**

**
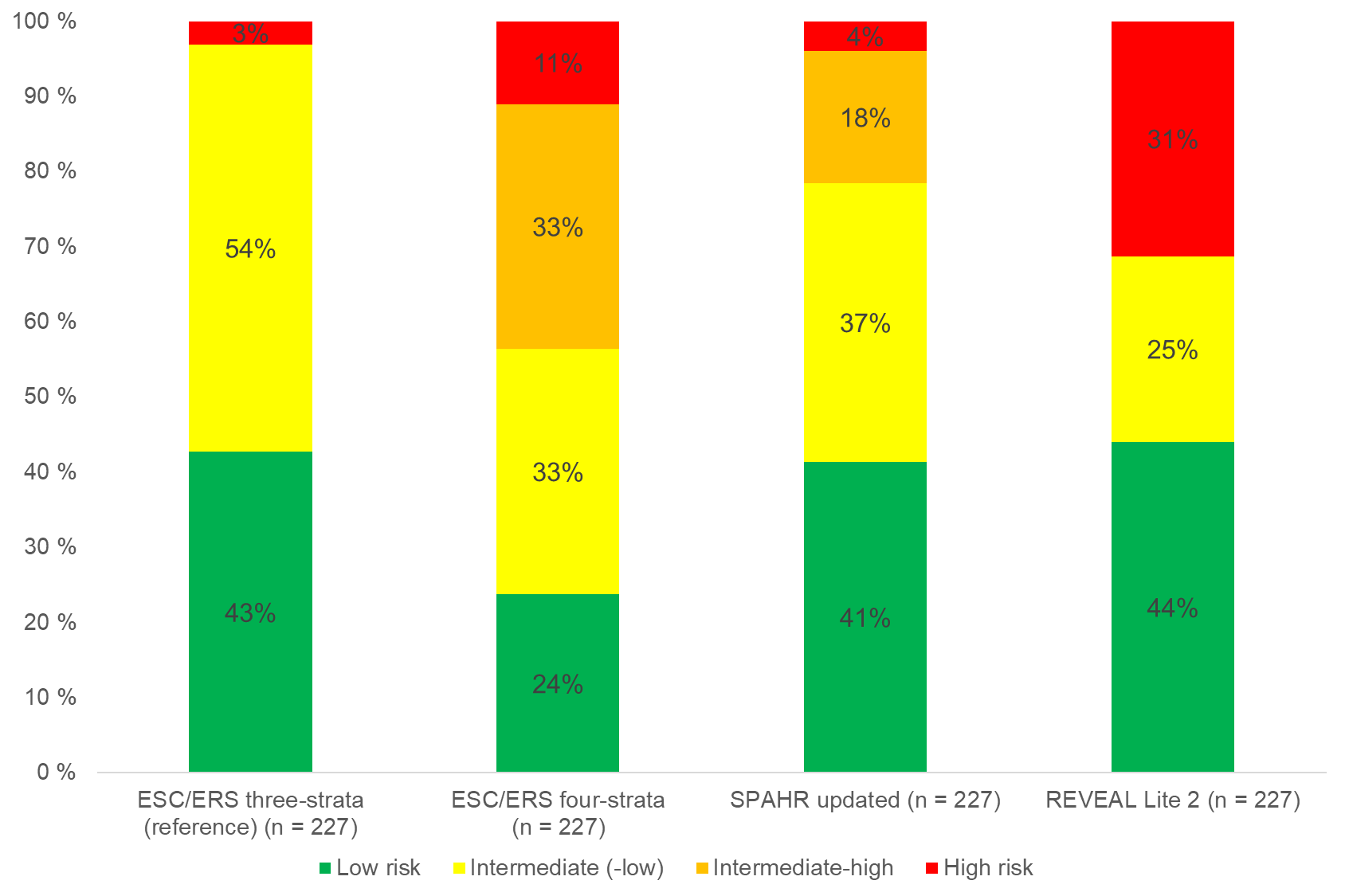
**

**B**

**
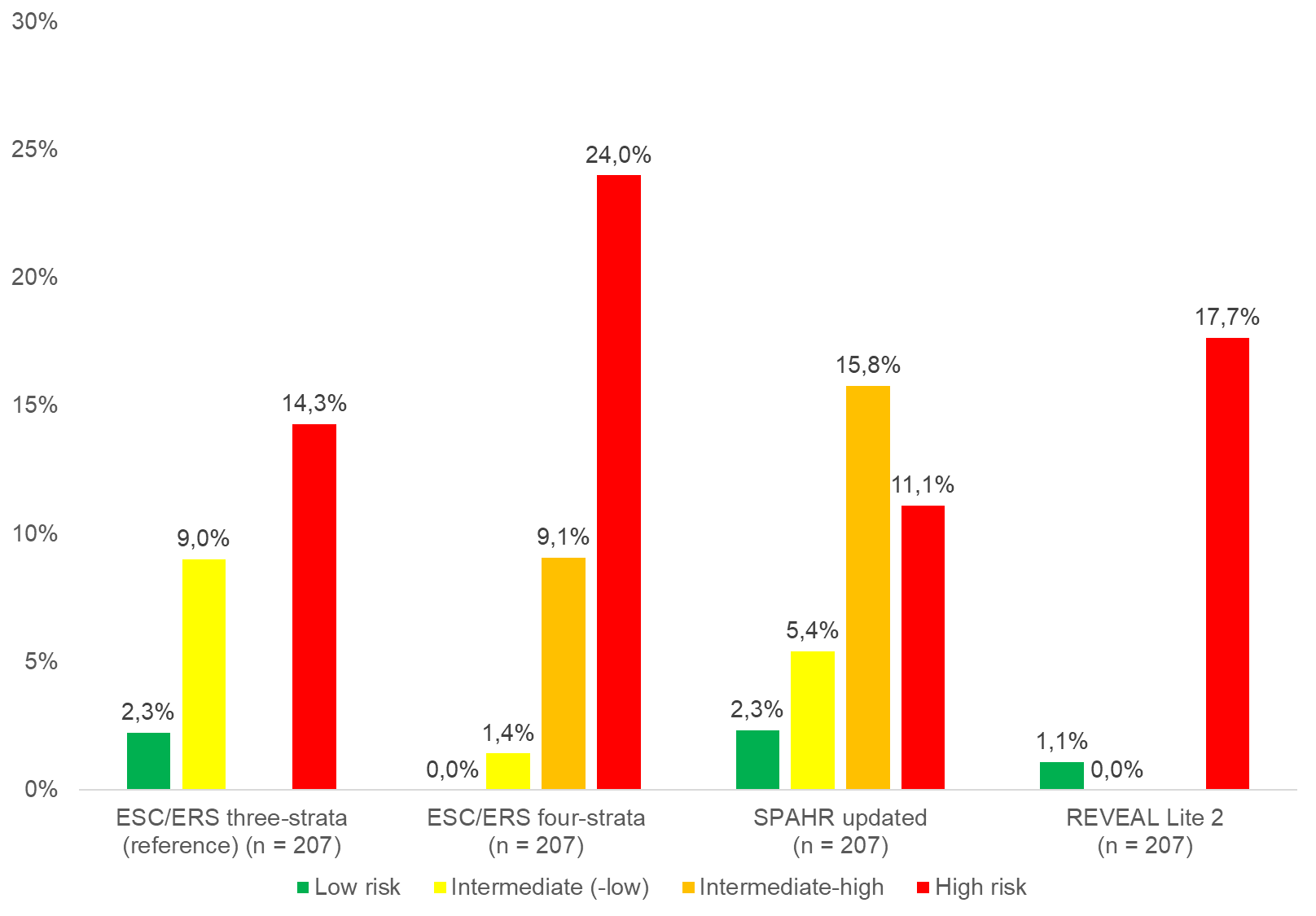
**

ESC/ERS: European Society of Cardiology and European Respiratory Society; SPAHR: Swedish Pulmonary Arterial Hypertension Registry; REVEAL: Registry to Evaluate Early and Long-Term PAH Disease Management. The 1-year mortality rate was determined for patients who were either deceased or had at least a one-year observation period.

**Supplementary Figure S11:** Transplant-free survival by risk groups in the four risk stratification tools in patients fulfilling all the four tools (n=227).


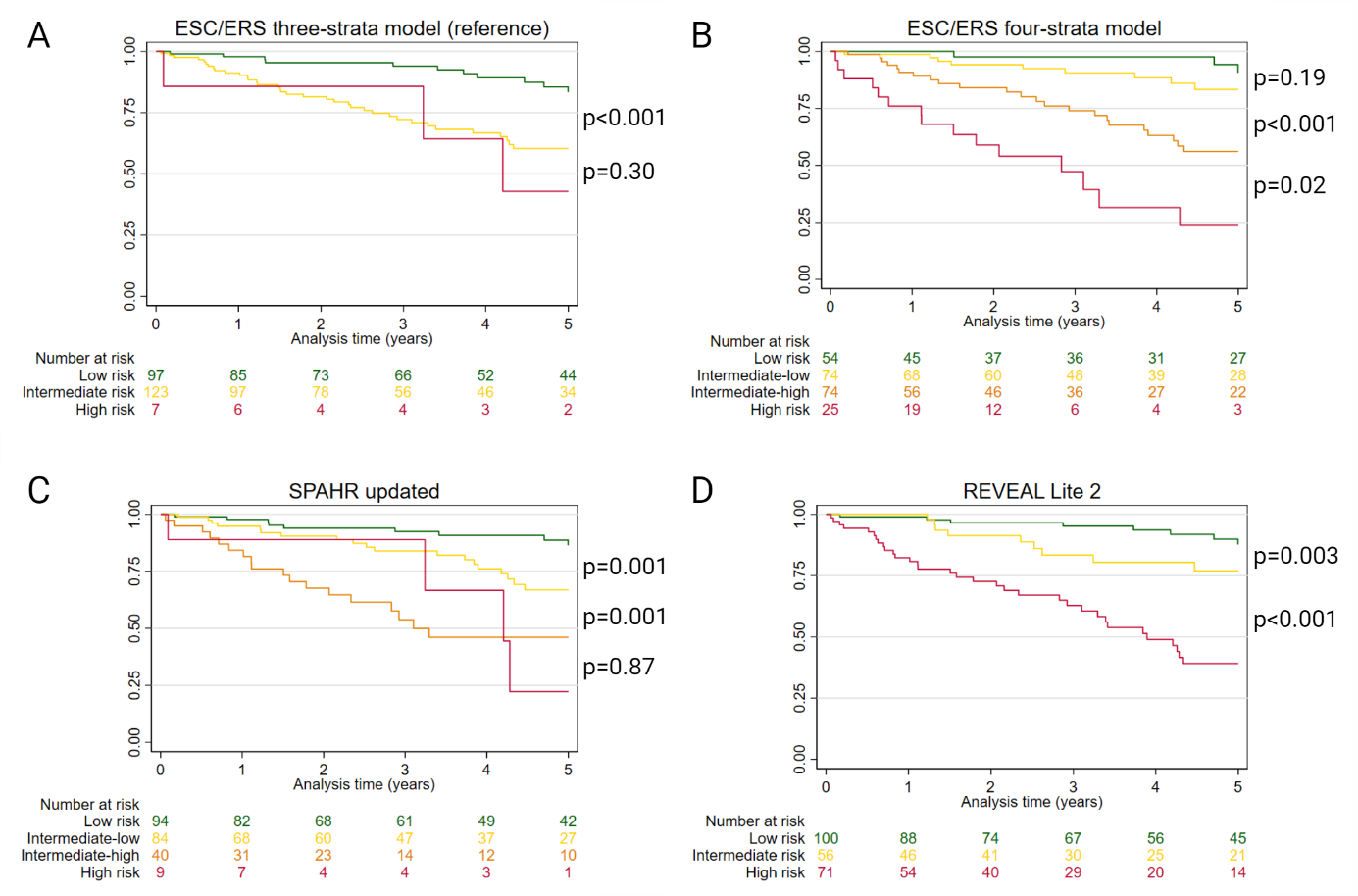


ESC/ERS: European Society of Cardiology and European Respiratory Society; SPAHR: Swedish Pulmonary Arterial Hypertension Registry; REVEAL: Registry to Evaluate Early and Long-Term PAH Disease Management.

**A**, ESC/ERS three-strata model; **B**, ESC/ERS four-strata model; **C**, “SPAHR updated”; and **D**, “REVEAL Lite 2”. P-values for pairwise comparison of the risk groups using the log-rank test.

**Supplementary Table S16:** Hazard ratios (HR) with 95% confidence intervals (CI) for all-cause mortality by risk groups in the four risk stratification tools in the subset of patients who fulfill all the four risk stratification tools.

|  | HR (95% CI), p-value | | | |
| --- | --- | --- | --- | --- |
|  | ESC/ERS three-strata model (reference) (n=227) | ESC/ERS four-strata model (n=227) | SPAHR updated (n=227) | REVEAL Lite 2 (n=227) |
| Intermediate  (-low) vs. low | 3.02  (1.82, 5.02) **<0.001** | 1.70  (0.73, 3.96)  *0.215* | 2.55  (1.42, 4.57)  **0.002** | 2.51  (1.30, 4.86)  **0.006** |
| Intermediate-high vs. low | N/A | 6.24  (2.85, 13.64)  **<0.001** | 6.23  (3.38, 11.50)  **<0.001** | N/A |
| High vs. low | 5.11  (1.91, 13.71)  **0.001** | 12.22  (5.22, 28.60)  **<0.001** | 6.76  (2.47, 18.51)  **<0.001** | 7.90  (4.45, 14.04)  **<0.001** |
| Intermediate-high vs. intermediate-low | N/A | 3.66  (2.03, 6.60)  **<0.001** | 2.45  (1.44, 4.15)  **0.001** | N/A |
| High vs. intermediate  (-low) | 1.69  (0.67, 4.26)  *0.263* | 7.17  (3.65, 14.07)  **<0.001** | 2.65  (1.02, 6.89)  **0.045** | 3.15  (1.80, 5.51)  **<0.001** |
| High vs. intermediate-high | N/A | 1.96  (1.10, 3.49)  **0.022** | 1.08  (0.41, 2.84)  *0.868* | N/A |

HR: hazard ratio; CI: confidence interval; AUC: area under the ROC curve; ESC/ERS: European Society of Cardiology and European Respiratory Society; SPAHR: Swedish Pulmonary Arterial Hypertension Registry; REVEAL: Registry to Evaluate Early and Long-Term PAH Disease Management; N/A: not applicable. P-values represent the significance of the hazard ratios obtained from univariable Cox regression analysis.

**Supplementary Table S17:** Performance of the risk stratification tools in predicting all-cause mortality compared to the ESC/ERS three-strata model (reference) in the subset of patients who fulfill all the four risk stratification tools (n=227).

| Comparison | Tool | AUC (95% CI) | *P* |
| --- | --- | --- | --- |
| ESC/ERS three-strata vs. ESC/ERS four-strata | ESC/ERS three-strata (reference) | 0.64 (0.57, 0.70) | **0.003** |
|  | ESC/ERS four-strata | 0.73 (0.66, 0.79) |  |
| ESC/ERS three-strata vs. SPAHR updated | ESC/ERS three-strata (reference) | 0.64 (0.57, 0.70) | **0.009** |
|  | SPAHR updated | 0.69 (0.62, 0.76) |  |
| ESC/ERS three-strata vs. REVEAL Lite 2 | ESC/ERS three-strata (reference) | 0.64 (0.57, 0.70) | **0.008** |
|  | REVEAL Lite 2 | 0.72 (0.65, 0.78) |  |

AUC: area under the ROC curve; CI: confidence interval; ESC/ERS: European Society of Cardiology and European Respiratory Society; SPAHR: Swedish Pulmonary Arterial Hypertension Registry; REVEAL: Registry to Evaluate Early and Long-Term PAH Disease Management.

Predictive abilities were evaluated using area under the ROC curve (AUC) based on univariable Cox regression analysis, and performance was compared to the ESC/ERS three-strata model (reference). P-values represent the statistical significance of differences in predictive performance between the risk stratification tools.

**Supplementary Figure S12:** Impact of risk stratification tools on predicting all-cause mortality in multivariable analysis in the subset of patients fulfilling all four risk stratification tools (n=227).


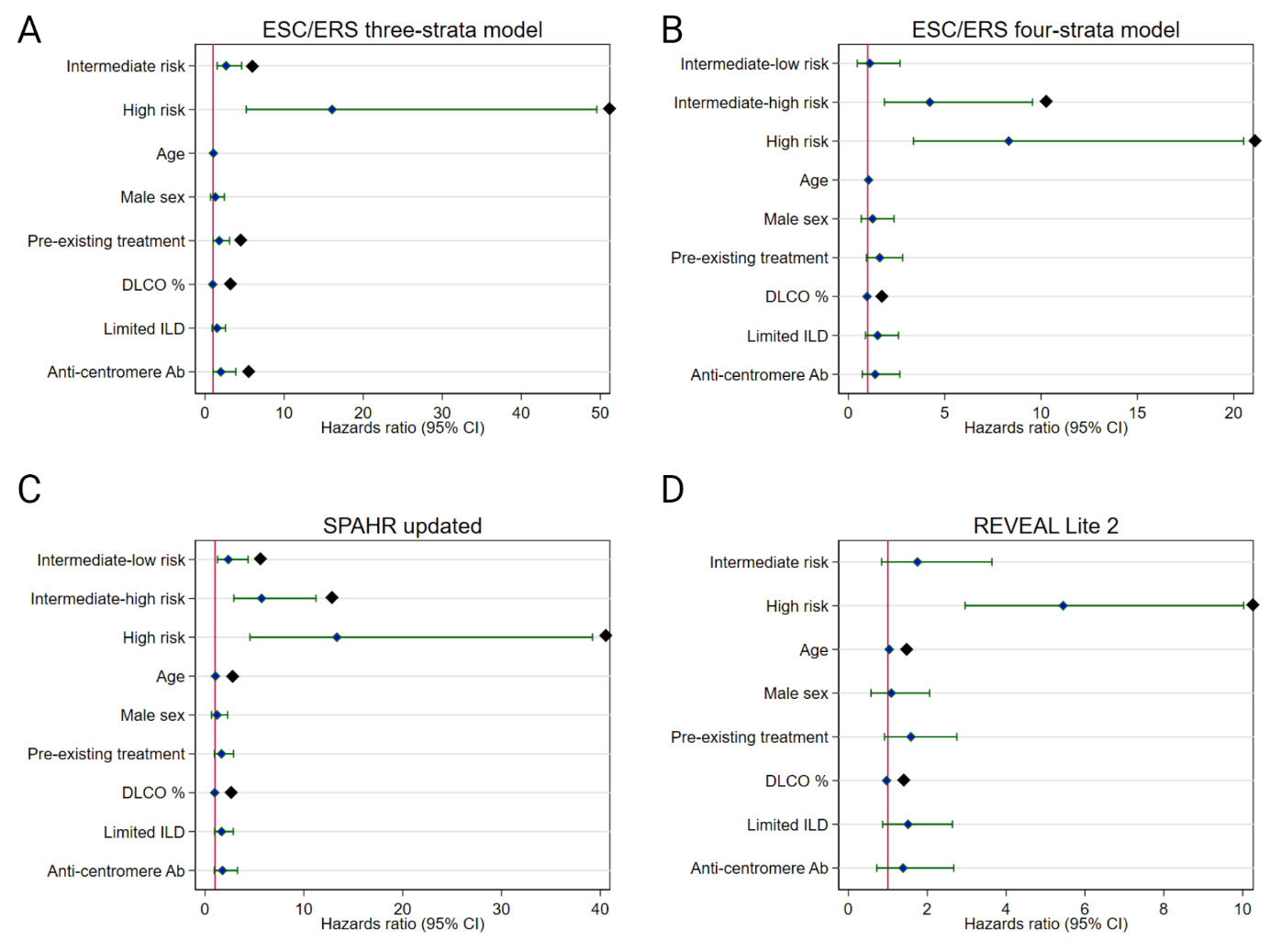


ESC/ERS: European Society of Cardiology and European Respiratory Society; SPAHR: Swedish Pulmonary Arterial Hypertension Registry; REVEAL: Registry to Evaluate Early and Long-Term PAH Disease Management; DLCO: diffusing capacity of the lung for carbon monoxide; ILD: interstitial lung disease, limited extent; Ab: antibodies; CI: confidence interval.

**A**, ESC/ERS three-strata model (reference); **B**, ESC/ERS four-strata model; **C**, “SPAHR updated”; and **D**, “REVEAL Lite 2”.

The multivariable models are adjusted for age, male sex, pre-existing vascular-targeted therapy, DLCO% predicted, ILD of limited extent, and anti-centromere antibodies, with hazard ratios (HR) and 95% confidence intervals (CI) shown for all variables. Hazard ratios of risk groups are referenced to the low-risk group. ♦= indicates p <0.05.

**Supplementary Table S18:** Performance of the adjusted risk stratification tools in predicting all-cause mortality compared to the corresponding univariable tool in the subset of patients fulfilling all four risk stratification tools (n=227).

| Risk stratification tool | AUC Univariable (95% CI) | AUC Multivariable (95% CI) | *P* |
| --- | --- | --- | --- |
| ESC/ERS three-strata | 0.64 (0.57, 0.71) | 0.70 (0.63, 0.77) | 0.052 |
| ESC/ERS four-strata | 0.72 (0.65, 0.79) | 0.73 (0.66, 0.80) | 0.708 |
| SPAHR updated | 0.69 (0.62, 0.76) | 0.72 (0.65, 0.79) | 0.248 |
| REVEAL Lite 2 | 0.71 (0.64, 0.78) | 0.73 (0.66, 0.80) | 0.433 |

ESC/ERS: European Society of Cardiology and European Respiratory Society; SPAHR: Swedish Pulmonary Arterial Hypertension Registry; REVEAL: Registry to Evaluate Early and Long-Term PAH Disease Management; AUC: area under the ROC curve; CI: confidence interval.

The risk stratification tools were adjusted for age, male sex, pre-existing vascular-targeted therapy, DLCO% predicted, ILD of limited extent, and anti-centromere antibodies. Predictive abilities were evaluated using area under the ROC curve (AUC), and performance was compared to the corresponding univariable tool. P-values represent the statistical significance of differences in predictive performance between the univariable and multivariable models.

**Supplementary Figure S13:** Impact of risk stratification tools on predicting all-cause mortality in multivariable analysis in the total SSc-PAH cohort using multiple imputation of missing covariates.


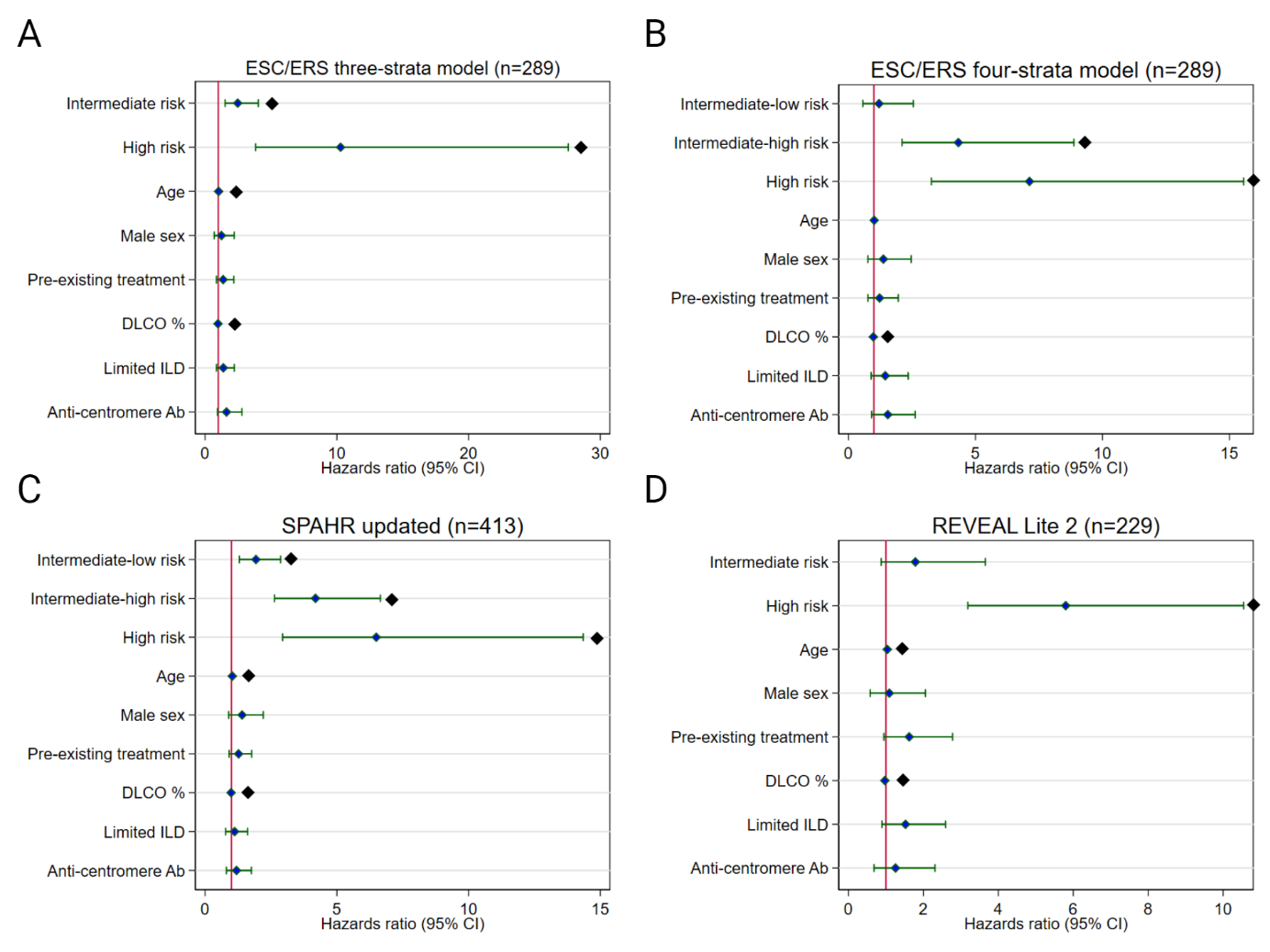


ESC/ERS: European Society of Cardiology and European Respiratory Society; SPAHR: Swedish Pulmonary Arterial Hypertension Registry; REVEAL: Registry to Evaluate Early and Long-Term PAH Disease Management; DLCO: diffusing capacity of the lung for carbon monoxide; ILD: interstitial lung disease, limited extent; Ab: antibodies; CI: confidence interval.

**A**, ESC/ERS three-strata model (reference); **B**, ESC/ERS four-strata model; **C**, “SPAHR updated”; and **D**, “REVEAL Lite 2”.

The multivariable models are adjusted for age, male sex, pre-existing vascular-targeted therapies, DLCO% predicted, ILD of limited extent, and anti-centromere antibodies, with hazard ratios (HR) and 95% confidence intervals (CI) shown for all variables. Hazard ratios of risk groups are referenced to the low-risk group. ♦= indicates p <0.05.

**Supplementary Table S19:** Performance of the adjusted risk stratification tools in predicting all-cause mortality compared to the corresponding univariable tool in the total SSc-PAH cohort using multiple imputation of missing covariates.

| Risk stratification tool | AUC Univariable (95% CI) | AUC Multivariable (95% CI) | *P* |
| --- | --- | --- | --- |
| ESC/ERS three-strata | 0.62 (0.56, 0.68) | 0.65 (0.59, 0.72) | 0.124 |
| ESC/ERS four-strata | 0.69 (0.63, 0.75) | 0.70 (0.64, 0.77) | 0.456 |
| SPAHR updated | 0.65 (0.60, 0.70) | 0.65 (0.60, 0.71) | 0.857 |
| REVEAL Lite 2 | 0.71 (0.64, 0.78) | 0.72 (0.65, 0.79) | 0.669 |

ESC/ERS: European Society of Cardiology and European Respiratory Society; SPAHR: Swedish Pulmonary Arterial Hypertension Registry; REVEAL: Registry to Evaluate Early and Long-Term PAH Disease Management; AUC: area under the ROC curve; CI: confidence interval.

The risk stratification tools were adjusted for age, male sex, vascular-targeted therapies, DLCO% predicted, ILD of limited extent, and anti-centromere antibodies. Predictive abilities were evaluated using area under the ROC curve (AUC), and performance was compared to the corresponding univariable tool. P-values represent the statistical significance of differences in predictive performance between the univariable and multivariable models.
